# Modulation of WNT and FGF18 enhances yield and subtype identity of hPSC-derived midbrain dopamine neurons

**DOI:** 10.1172/JCI190954

**Published:** 2026-05-15

**Authors:** Tae Wan Kim, Jinghua Piao, Vittoria D. Bocchi, So Yeon Koo, Se Joon Choi, Fayzan Chaudhry, Donghe Yang, Hyein S. Cho, Emiliano Hergenreder, Lucia Ruiz Perera, Subhashini Joshi, Zaki Abou Mrad, Nidia Claros, Shkurte Ademi Donohue, Yeong Eun Im, Hyo Jae Jeong, Anika K. Frank, Ryan M. Walsh, Eugene V. Mosharov, Doron Betel, Viviane Tabar, Lorenz Studer

**Affiliations:** 1 Center for Stem Cell Biology and; 2 Developmental Biology Program, Memorial Sloan-Kettering Cancer Center, New York, New York, USA.; 3 Department of New Biology and; 4 Department of Biomedical Science and Engineering, Daegu Gyeongbuk Institute of Science and Technology (DGIST), Daegu, South Korea.; 5 Department of Neurosurgery and; 6 Cancer Biology and Genetics Program, Memorial Sloan-Kettering Cancer Center, New York, New York, USA.; 7 Neuroscience program, Graduate School of Medical Sciences, Weill Cornell Medical College, New York, New York, USA.; 8 Department of Psychiatry, Columbia University Medical Center, New York, New York, USA.; 9 Institute for Computational Biomedicine, Division of Hematology/Oncology, Department of Medicine, Weill Cornell Medical College, New York, New York, USA.; 10 Tri-Institutional Ph.D. program in Computational Biology, New York, New York, USA.

**Keywords:** Development, Neuroscience, Neurodevelopment, Parkinson disease, Stem cell transplantation

## Abstract

While clinical trials of human pluripotent stem cell–derived midbrain dopamine (mDA) neuron precursor grafts for Parkinson’s disease (PD) are ongoing, current protocols remain suboptimal. In particular, the yield of TH+ mDA neurons after in vivo grafting and the expression of certain mDA neuron and subtype-specific markers require improvement. Single-cell transcriptomic analyses of grafts have revealed low proportions of mDA neurons and substantial off-target contamination. Here, we present an optimized mDA neuron differentiation strategy that builds on our clinical-grade (“Boost”) protocol by adding FGF18 and IWP2 treatment (“Boost+”) at the neurogenesis stage. Boost+ mDA neurons show higher expression of EN1, PITX3, and ALDH1A1. Improvements in mDA neuron yield and transcriptional similarity to primary mDA neurons are observed in vitro and following transplantation. Single-nucleus RNA sequencing demonstrates enrichment of A9 mDA neurons within Boost+ grafts. Functional studies in vitro demonstrate increased dopamine production and release and improved electrophysiological properties. In vivo analyses show higher percentages of TH+ mDA neurons, resulting in efficient rescue of amphetamine-induced rotation behavior in the 6-OHDA rat model and rescue of deficits in some nondrug-induced assays, including the ladder rung assay, which are not improved by Boost mDA neurons. The Boost+ conditions present an optimized differentiation protocol with advantages for disease modeling and mDA neuron grafting paradigms.

## Abstract 2

This study introduces a refined method for producing dopamine neurons in vitro, providing a new protocol for applications in cell therapy for Parkinson's disease.

## Introduction

Parkinson’s disease (PD) is the second most common neurodegenerative disease, characterized by the progressive degeneration of midbrain dopamine (mDA) neurons, resulting in motor symptoms such as tremor, gait imbalance, and bradykinesia (1). Cell replacement therapy via transplantation of mDA neuron precursors presents a promising strategy to reverse motor dysfunction in PD both at the cellular and circuit levels (2, 3). Human pluripotent stem cells (hPSCs), both human embryonic stem (ES) and induced pluripotent stem (iPS) cells, provide a scalable source for generating mDA neurons (4, 5). Grafting hPSC-derived mDA neurons has shown success in preclinical animal models of PD (6–9) and moved from preclinical to clinical translation in PD patients (10–15). Despite such rapid progress, mDA neuron yield remains suboptimal, with only around 10% of total cells within the graft expressing tyrosine hydroxylase (TH), the rate-limiting enzyme for dopamine synthesis (10–12, 16–18). In most studies, hPSC-derived mDA precursor grafts yield a mixture of cells, which include off-target populations, including subthalamic and hindbrain neurons, astrocytes and oligodendroglia, and nonneural cells such as vascular leptomeningeal-like cells (VLMCs) (4).

Early work demonstrated mDA neuron derivation via a midbrain floor plate intermediate (19, 20). Several groups have further optimized floor plate-based mDA neuron derivation by modifying timing and duration of patterning factors modulating WNT, SHH, and FGF8 signaling. However, considerable variability remains in mDA neuron yield and the presence of potential off-target populations (4). Common mDA neuron markers FOXA2 and LMX1A are also expressed by anterior, subthalamic precursor lineages (9, 21). EN1 demarcates the midbrain from anterior, diencephalic domains (22), and EN1 expression is a predictor of graft outcome (9). However, EN1 extends into the anterior hindbrain. OTX2 marks midbrain but not hindbrain anlage, and the quadruple expression of FOXA2+LMX1A+EN1+OTX2+ defines the desired precursor population. Previous work reported that late administration of FGF8b during mDA differentiation leads to improved EN1 expression in FOXA2+LMX1A+ precursors and reduces anterior off-target cells (9). However, protocols applying FGF8b have been shown to also generate VLMC-like cell types, expressing fibroblast makers such as COL1A1 and PDGFR in vitro or after transplantation in vivo (23, 24).

The derivation of substantia nigra pars compacta (SNc) A9 mDA neurons is particularly important for cell-based therapy in PD, given their selective loss in PD. Mouse developmental studies identified ALDH1A1 as a key A9 mDA neuron marker (25) within the SOX6+ lineage, marking a subset of SNc mDA neurons highly vulnerable in PD (26). Several recent studies have characterized mDA neuron diversity during development in vivo (21, 25, 27, 28). However, there is a paucity of single-cell data from hPSC-derived mDA neurons after grafting. Early studies showed that single-cell RNA-seq (scRNA-seq) of hPSC-derived mDA precursor grafts captures a very low yield of mDA neurons, representing less than 7% of the total cells sequenced with more than 90% off-target cells including VLMCs (24). The scRNA-seq–based mDA neuron proportion differs significantly (up to 10-fold) from the percentage of mDA neurons reported by histological data (24), suggesting low efficacy of neuron capture by scRNA-seq due to selective loss during enzymatic and mechanical dissociation (29). A rigorous single-cell framework for assessing lineage fidelity, subtype identity, and off-target populations in hPSC-derived grafts is therefore essential.

Here, we present a protocol called Boost+, based on our previously reported Boost differentiation conditions (6), which was the foundation of our clinical mDA product. Boost+ includes additional treatment with FGF18 at the stage of neurogenic differentiation to maintain EN1 expression without significantly inducing VLMC-like lineages. Concomitant treatment with the WNT inhibitor IWP2 enhances mDA neuron marker expression, reduces off-target markers, and triggers increased ALDH1A1 expression. The resulting Boost+ mDA cell product yields a higher portion of TH+ mDA neurons in vitro and elevated expression of EN1 and PITX3. Furthermore, Boost+ patterned mDA neurons exhibit improved electrophysiological function and increased dopamine release. Using single-nucleus RNA-seq, we demonstrate a marked enrichment of mDA neurons within Boost+ grafts and an increased proportion of ALDH1A1^+^ mDA neurons closely resembling primary human A9 mDA neurons. Both Boost and Boost+ grafts rescue drug and nondrug-induced PD behaviors in the 6OHDA lesion rat model, but Boost+ grafts induce recovery in additional assays including the ladder rung test.

## Results

### FGF18 and IWP2 at neurogenic conversion induce improved yield of mDA neurons.

We recently reported on the Boost protocol to derive mDA neurons suitable for clinical translation involving the biphasic activation of WNT signaling and resulting in robust EN1 levels at day 11 of differentiation (6). However, EN1 expression in the Boost protocol decreases by day 16 (Supplemental Figure 1A; supplemental material available online with this article; https://doi.org/10.1172/JCI190954DS1). In contrast, EN1 remains highly expressed in primary mDA lineages (30, 31) and FGF signaling is required to sustain its expression (32). Several groups have used FGF8b treatment during either early or late stages of mDA neuron differentiation (9, 19). In the Boost protocol, exogenous FGF8b is not required for EN1 induction at the floorplate stage, as WNT-Boost triggers endogenous FGF8b expression and expression of FGF8-dependent genes such as *PAX2*, *PAX5*, and *PAX8* (6). In contrast, late FGF8b during neurogenic differentiation maintained EN1 (Supplemental Figure 1, B and C) but also induced off-target markers such as SIX1 and SMA, a finding highly dependent on the onset and duration of FGF8b treatment (Supplemental Figure 1, B and C). To overcome this trade-off, we tested an alternative ligand FGF18 (33), which unlike FGF8b, is selectively expressed in the midbrain (34). Both FGF8b and FGF18 significantly increased EN1 expression, similar to a recent study testing the ability of FGF17 and FGF8 to induce EN1 (35). However, FGF18 showed reduced SIX1, SMA, and COL1A1 induction compared to FGF8b treatment (Supplemental Figure 1, D–F).

**Figure 1:**
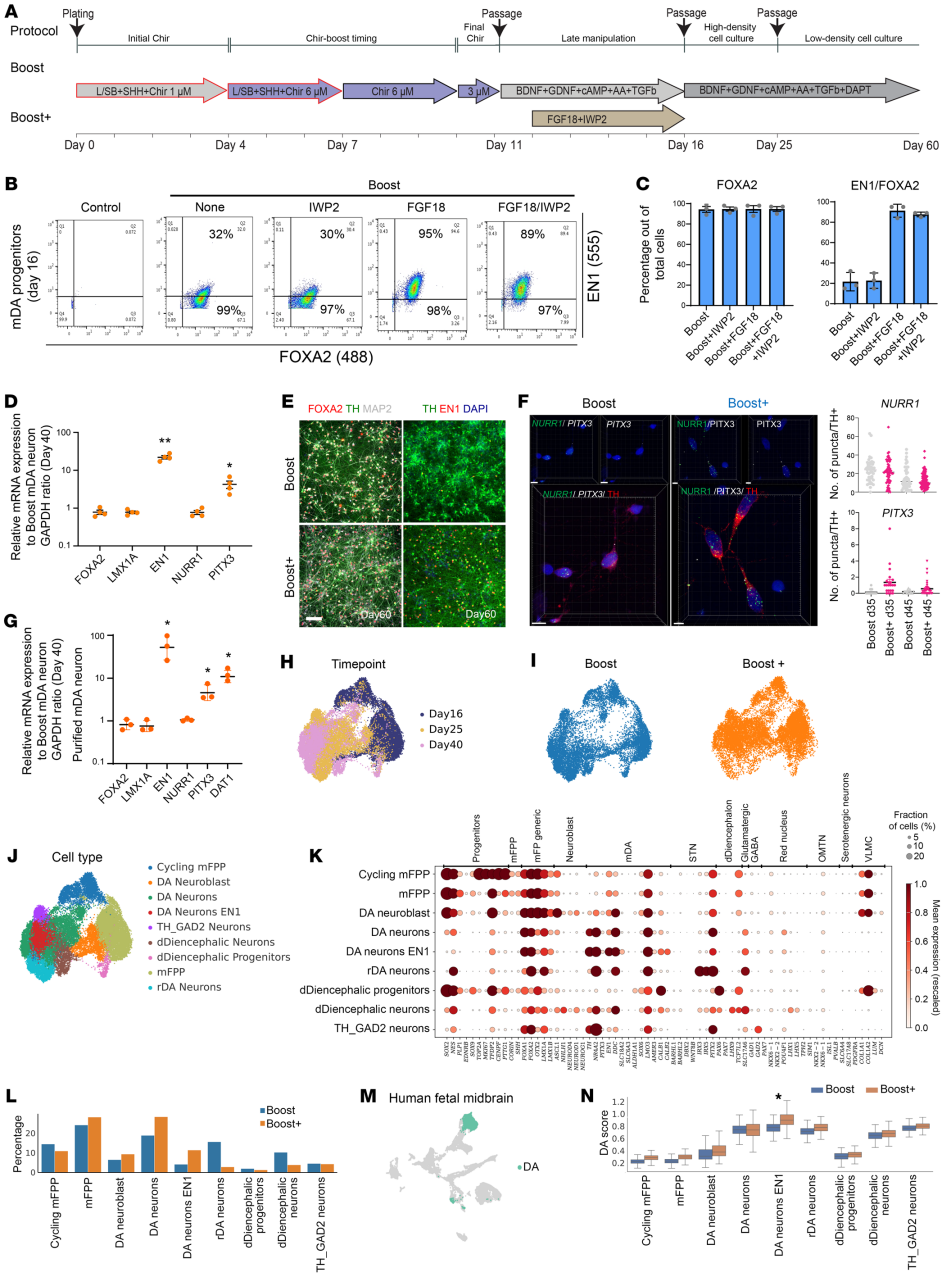
FGF18 and IWP2 at neurogenic conversion induce improved yield of mDA neurons. (**A**) Schematic illustration of the Boost and Boost+ mDA differentiation method. (**B**) Representative flow cytometry dot plots showing intracellular expression of FOXA2 and EN1 in day 16 progenitors differentiated under the indicated conditions. IWP2 and/or FGF18 were treated from day 12 to 16. (**C**) Quantification of FOXA2^+^ and FOXA2^+^EN1^+^ populations across differentiation conditions (*n* = 3; Mean ± SEM) from **B**. (**D**) qRT-PCR analysis of mDA neurons at day 40 for mDA neuron markers derived from the Boost and Boost+ methods (*n* = 4; Mean ± SEM). *P < 0.05, **P < 0.01. (**E**) Representative immunofluorescence images showing expression of mDA neuron markers FOXA2, TH, and EN1 in day 60 neurons. (**F**) Representative confocal image of RNA FISH of day 45 mDA neurons, colabeled with TH, mature mDA markers (left). Human RNA probes against *NURR1* and *PITX3* were used. The number of RNA FISH dots for each RNA gene (*NURR1* and *PITX3*) was quantified per TH+ mDA neurons at day 35 and 45 (right). *n* = 4, 2-tailed Student’s *t* test, *P < 0.05. Scale bars: 100 μm (**E**), 7 μm (**F** left), 5 μm (**F** right). (**G**) qRT-PCR analysis of homogeneous postmitotic mDA neurons purified at day 40 using CD49e and CD184 for mDA markers in the Boost and Boost+ conditions (*n* = 3; Mean ± SEM). *P < 0.05. (**H**–**J**) UMAP plots of in vitro scRNA-seq data colored by timepoint (**H**), protocol (**I**), and cell type (**J**). (**K**) Dotplot of canonical marker genes of midbrain progenitors, DA neuron subtypes, and off target cells. (**L**) Overall percentage of cell types across protocols. (**M**) UMAP of fetal midbrain dopamine (DA) neurons (49), selected to determine highly specific markers. (**N**) Boxplots showing the distribution of enrichment scores for each cell type and protocol according to fetal dopamine neurons. Mann-Whitney rank test and Benjamini-Hochberg correction; ***P < 0.001.

While WNT activation is essential for EN1 induction, extended canonical WNT signaling may interfere with mDA neurogenesis (36–38). Accordingly, we conducted studies using the porcupine inhibitor IWP2 to trigger timed abrogation of both canonical and noncanonical WNT signaling. Flow analysis, immunofluorescent staining, and RT-qPCR demonstrated that adding IWP2 together with FGF18 during neurogenic conversion (called Boost+) leads to a high percentage of quadruple FOXA2+LMX1A+OTX2+EN1+ cells at day 16 comparable to FGF18 alone (Figure 1, A–C, and Supplemental Figure 1, G and H). However, cotreatment with FGF18 and IWP2 induced increased ALDH1A1 expression (Supplemental Figure 1H). Boost+ was similarly effective across additional hPSC lines, including MEL1 hESCs and J1 iPSCs (Supplemental Figure 1, I and J). At postmitotic stages, Boost+ mDA neurons exhibited higher levels of key identity markers, including EN1 and PITX3 (Figure 1, D and E). Quantitative RNA FISH and studies on purified mDA neurons based on surface marker expression (39) confirmed higher expression of *PITX3* and dopamine transporter (DAT) in Boost+ versus Boost mDA neurons (Figure 1, F and G, and Supplemental Figure 2I).

We next performed scRNA-seq at days 16, 25, and 40 under Boost and Boost+ conditions (Figure 1, H and I) and classified cell clusters using canonical markers (Figure 1, J and K, and Supplemental Figure 2A). At day 16, cultures comprised ventral floor plate progenitors (Supplemental Figure 2B), progressed to mixed neuroblast–neuron populations by day 25 (Supplemental Fig. 2C), and neuronal lineages by day 40 (Supplemental Figure 2D). Cell-cycle analysis revealed a higher proportion of cycling cells in the Boost versus Boost+ protocol at day 16 (Supplemental Figure 2E). Expression of *TH*, *NR4A2*, and low expression of *PITX3* defined mDA neurons *PITX3* with a subset expressing *EN1* (Figure 1K). *TH^+^EN1^+^* mDA neurons increased approximately 3-fold under Boost+ by day 40 (15% to 47%; Supplemental Figure 2D). Conversely, a rostral DA population (rDA neuron) expressing *TH*, *NR4A2*, *IRX2*, *IRX5,* and *PITX2* was enriched under Boost conditions (Figure 1, K and L, and Supplemental Figure 2, A, C, and D). A small off-target population was identified expressing dorsal diencephalic markers of prethalamic (*PAX6*, *LHX2*, *LHX9*, *LEF1*, *TCF7L2*, *SLC17A6*) (40–44) and pretectal fates (*MEIS2*, *LHX1*, *BARHL2*, *TBR1*) (41, 43, 45). Furthermore, we observed a small population of *TH* and *GAD2-*expressing cells that could represent an immature *TH^+^GAD2^+^EBF2^+^CALCRL^+^* population (Figure 1K and Supplemental Figure 2, A–D) recently discovered (46) within the adult midbrain. We did not detect subthalamic nucleus (STN), red nucleus, or oculomotor neurons (OMNT). Although some COL1A1+ cells were observed in the Boost+ (Supplemental Figure 2, G and H), bona fide VLMCs were not identified based on lack of *PDGFRA* expression (Figure 1K and Supplemental Figure 2A).

Lineage markers did not fully segregate, with some cells coexpressing mDA markers (*TH*, *NR4A2*, *EN1*) and subthalamic markers such as *PITX2* or *POU4F1*. These hybrid states likely reflect in vitro stress responses, as reported in organoids (47), which impede lineage resolution. Nevertheless, Boost+ increased mature *TH*^+^*EN1*^+^ mDA neurons threefold and reduced rostral and diencephalic off-targets from 22% (Boost) to 8% (Boost+) (Figure 1L, and Supplemental Figure 2F). To assess mDA neuron fidelity, we scored in vitro–derived cells against a 100-gene signature of human fetal mDA neurons (48) (Figure 1M). *TH^+^EN1^+^* neurons generated under Boost+ achieved the highest similarity to primary fetal mDA neurons (Figure 1N).

### Improved dopaminergic function of Boost+-derived mDA neurons in vitro.

**Figure 2:**
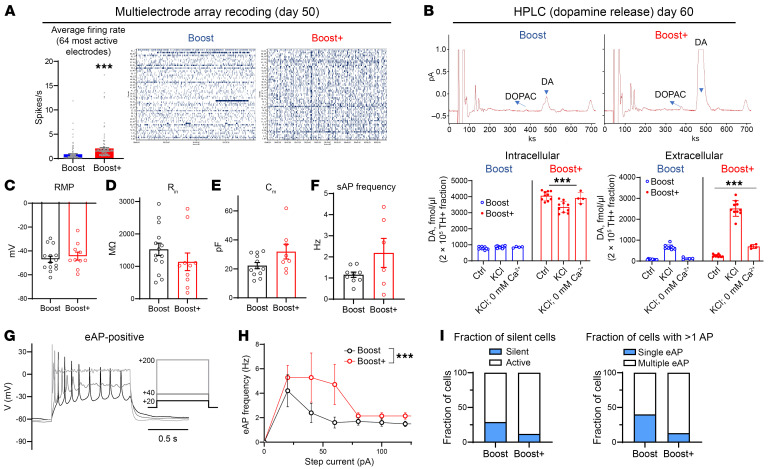
Improved dopaminergic function of Boost+ mDA neurons in vitro. (**A**) High-density multielectrode array recordings reveal increased firing rates at day 50 in mDA neuron derived from Boost+ versus Boost protocols. Left, mean firing rates calculated from 60 s of activity in the 1 of 64 most active electrodes of each probe (*n* = 256 electrodes from 4 MEA probes). Right, representative spike raster-gram displaying 1 m MEA recordings. Data are represented as mean ± SD. ***P < 0.001. (**B**) Representative traces (top) and statistical analysis (bottom) of HPLC recording of DA release evoked by 80 mM KCl stimulation (5 min). DA concentration in fmol per 2 × 10^5^ cells divided by the fraction of TH+ mDA neurons (0.25 in Boost and 0.41 in Boost+. Stimulation in the absence of extracellular Ca^2+^ is used as a control. ***P < 0.001 from all other groups by 1-way ANOVA (*n* = 10 culture dishes for control and KCl groups and *n* = 4 for KCl in 0 mM Ca^2+^). (**C**–**F**) Electrophysiological characteristics of cultured mDA neurons at day 60, including resting membrane potential (**C**, *n* = 14 and 10 cells), input resistance (**D**, *n* = 14 and 10), membrane capacitance (E, *n* = 12 and 8), and spontaneous AP frequency (F, *n* = 10 and 6). (**G**) Examples of mDA neuron responses to current injections. (**H**) Dependence of spike frequency evoked by current injection (eAP) on the current amplitude. ***P < 0.001 by 2-way ANOVA. (**I**) The fraction of neurons responding to current injections was higher in the Boost+ protocol (*P* < 0.001 by χ^2^ test).

We measured the emergence of spontaneous in vitro network activity in mDA neurons at day 50 using a high-density micro-electrode array (MEA) containing 4,096 electrodes. Boost+-derived mDA neurons showed higher levels of spontaneous activity than Boost (Figure 2A). In addition, high-performance liquid chromatography (HPLC) analysis of TH+ mDA neurons at day 60 in the Boost+ protocol showed a 3–5-fold increase in dopamine levels upon KCl or Ca^2+^ stimulation (Figure 2B). Basal membrane properties of mDA neurons (Boost+ versus Boost) by patch-clamp recording on day 60 showed no major differences, though Boost+ mDA neurons showed a trend toward lower input resistance, higher cell capacitance, and an increased spontaneous action potential (sAP) frequency (Figure 2, C–F). Upon current injections, Boost+ mDA neurons showed an increased evoked action potential (eAP) frequency upon depolarization, maintained stimulated firing activity for a longer time, and had a higher fraction of responding neurons than in Boost protocol (Figure 2, G–I).

### In vivo cell type composition by single nucleus sequencing.

**Figure 3:**
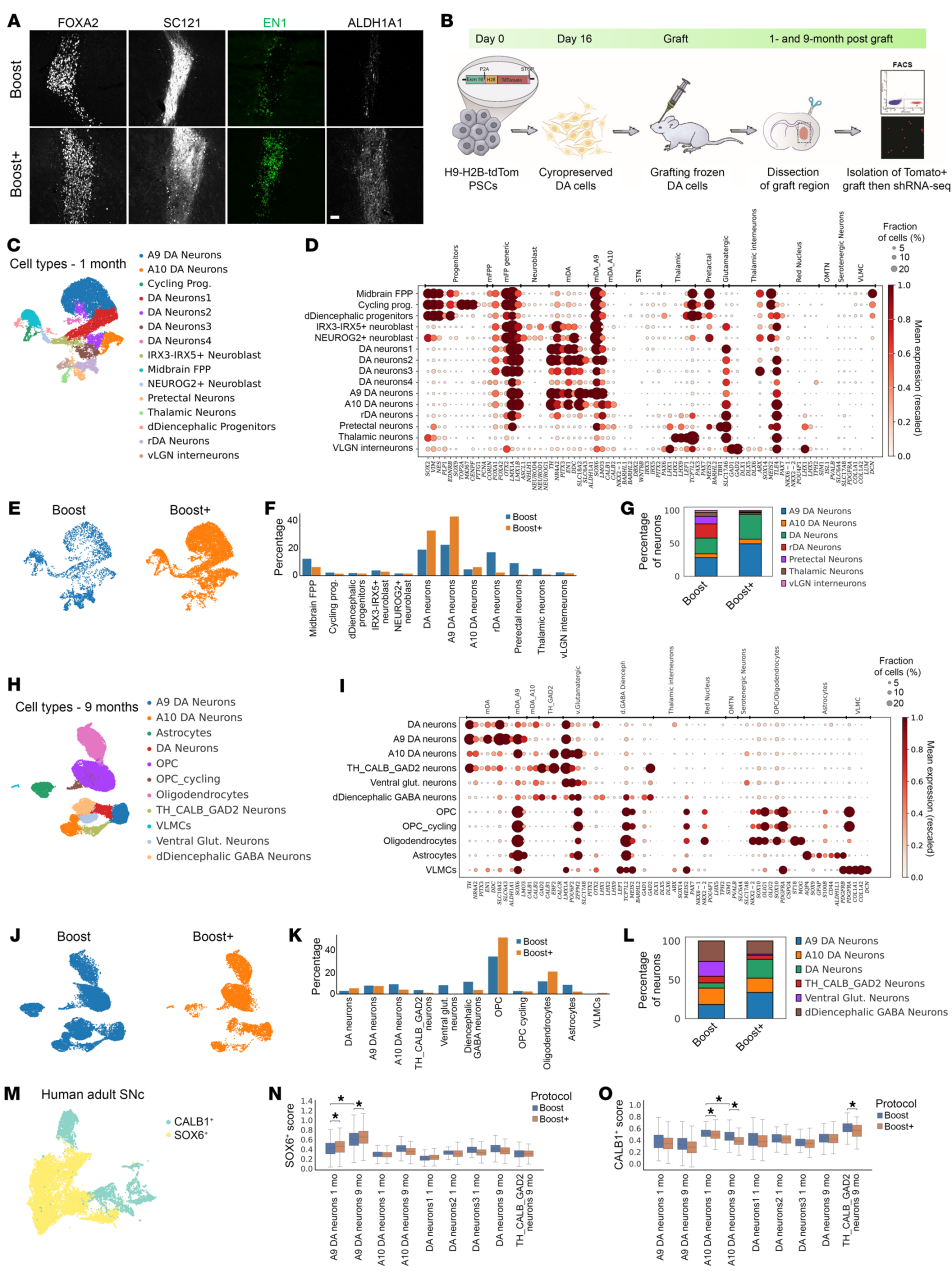
In vivo cell type composition by snRNA-seq. (**A**) Representative microscopy images of 1-month-old (equivalent of day 45 differentiation in vitro) intrastriatal grafts from Boost versus Boost+ patterned progenitors (day 16) on various markers, FOXA2, SC121, EN1, and ALDH1A1. (**B**) Schematic illustration of the snRNA-seq from the grafts 1- and 9-months postimplantation of the mDA neuron progenitor (day 16) derived from the Boost and Boost+ method. (**C**) UMAP plot of 1-month grafted cells from the Boost and Boost+ protocols, colored by cell types. (**D**) Dotplot of canonical marker genes of midbrain progenitors, mDA neuron subtypes, and off-targets cells (**E**) UMAP plot colored by protocol at 1 month. (**F** and **G**) Percentages of each cell type colored by protocol (**F**) and different neuronal subtypes in each protocol at 1 month (**G**). (**H**) UMAP plot of 9 months grafted cells from the Boost and Boost+ protocols, colored by cell types. (**I**) Dotplot of canonical marker genes of midbrain progenitors, mDA neuron subtypes, and off-target cells at 9 months. (**J**) UMAP plot, colored by protocol at 9 months. (**K** and **L**) Percentages of each cell type, colored by protocol (**K**) and different neuronal subtypes in each protocol at 9 months (**L**). (**M**) UMAP of adult midbrain dopamine (mDA) neurons, divided by SOX6-and CALB1-positive neurons that were selected to determine highly specific markers for A9 and A10 DA neurons, respectively. (**N** and **O**) Boxplots showing the distribution of enrichment scores for each cell type, protocol, and time point according to A9 (**N**) and A10 (**O**) mDA neurons of adult signatures. Mann-Whitney rank test and Benjamini-Hochberg correction; *P < 0.001.

To assess how the Boost+ protocol changes cell type composition in vivo, we transplanted day 16 mDA precursors from Boost or Boost+ protocol into the striatum of adult NSG mice. At 1 month after implantation, histological analysis revealed that Boost+ grafts contained more EN1^+^ and ALDH1A1^+^ mDA neurons, while FOXA2 expression was comparable (Figure 3A). To resolve graft composition in detail, we performed snRNA-seq on TdTomato-labeled Boost and Boost+ grafts 1 month after transplantation (Figure 3B and Supplemental Figure 3A). The snRNA-seq data showed more conclusive mDA neuronal subtype identities, including A9 mDA neurons expressing *ALDH1A1*, *SOX6,* and *LMO3*; A10 DA neurons expressing *CALB1* and *CALB2* (Figure 3, C and D); and rostral DA neurons expressing low levels of *PITX2* together with *TH* and *NR4A2*. All mDA neurons expressed canonical mDA markers, including *TH*, *NR4A2*, *PITX3*, *EN1,* and *DDC* in combination with the dopamine transporters *SLC18A2* and *SLC6A3* (Figure 3, C and D, and Supplemental Figure 3B), implying a higher degree of maturity in grafted mDA neurons.

In addition to abundant mDA neurons, we identified minor off-target populations, including thalamic and pretectal neurons defined by *LHX2*, *LHX9*, *LEF1*, *TCF7L2* or *LHX1*, *LHX9*, *MEIS2*, *BARHL2*, and a small inhibitory group resembling interneurons of the ventral lateral geniculate nucleus, coexpressing *GAD1*, *GAD2*, *DLX1*, *DLX5*, *DLX6*, *OTX2*, and *TLE4* (Figure 3, C and D, and Supplemental Figure 3B). Partition-based graph abstraction (PAGA) analysis linked midbrain floor plate progenitors to mDA neurons and dorsal diencephalic progenitors to off-target diencephalic neurons, suggesting these progenitors give rise to the respective fates (Supplemental Figure 3C). We did not detect bona fide VLMCs (49) as defined by coexpression of *PDGFRA*, *COL1A1*, *COL1A2*, *LUM*, and *DCN* (Figure 3D and Supplemental Figure 3, B–E). Consistent with in vitro findings, Boost+ suppressed diencephalic off-targets (18%–5%) and increased mDA neurons (46%–82%) relative to Boost (Figure 3, E–G).

Boost+ generated approximately twice the fraction of highly specific *SLC6A3^+^TH^+^* and A9 *SLC6A3^+^ALDH1A1^+^* mDA neurons (Supplemental Figure 3, E–G). To independently validate A9 enrichment, we subset the snRNA-seq data for *TH* and *NR4A2* and subclustered these cells (Supplemental Figure 3, H and I), confirming a higher proportion of *ALDH1A1^+^* neurons under Boost+ (Supplemental Figure 3, J and K). KEGG analysis showed enrichment of dopamine signaling pathways in the *ALDH1A1* compartment, whereas *CALB1^+^* neurons were enriched for morphine addiction pathways (Supplemental Figure 3L). Both Boost and Boost+ protocols yielded A9 mDA neurons upon transplantation (clusters 0 and 1; A9-like cells in Supplemental Figure 3M). However, A9 neurons under Boost+ expressed significantly higher levels of *TH*. Gene Ontology analysis revealed enrichment of glycolytic and ATP metabolic pathways in Boost+ A9-like cells.

To examine long-term maturation, we analyzed grafts at 9 months after transplantation (Figure 3, H–J, and Supplemental Figure 4A). This revealed a marked expansion of glial populations, particularly OPCs and oligodendrocytes (Figure 3K), consistent with progressive gliogenesis in grafts over time (23, 50, 51). Notably, the abundance of oligodendrocytes is a feature more commonly associated with fetal grafts (23). At 9 months, both A9 and A10 mDA subtypes were present, with A9 neurons remaining more abundant in Boost+ (33.6%) than Boost (18.1%). Conversely, A10 neurons were slightly enriched in Boost (21.1%) relative to Boost+ (18.5%). While the proportion of SLC6A3^+^TH^+^ neurons no longer differed (Supplemental Figure 4B), SLC6A3^+^ALDH1A1^+^ A9-like neurons remained significantly enriched in Boost+ (Supplemental Figure 4C). Importantly, diencephalic off-target populations were less obvious in the 9-month versus 1-month data, suggesting a progressive loss of subtype identity over time, potentially due to the striatal host environment (Figure 3I and Supplemental Figure 4A). Off-target populations remained substantially reduced in the Boost+ condition, with ventral glutamatergic neurons decreasing from 19.1% to 1.5%, and dorsal diencephalic GABAergic neurons from 26.4% to 17.0% (Figure 3L). At 9 months, a very small VLMC population (approximately 1%) emerged in Boost+ grafts. These cells likely originate from floor plate progenitors expressing *COL1A1* at 1 month (6% within the FPP cluster; Supplemental Figure 4D), consistent with reports that some progenitors retain multipotency to generate fibroblast-like cells after engraftment (51).

To test subtype authenticity, we compared our data to adult mDA neurons. First, we defined A9 (*SOX6* positive neurons) and A10 (*CALB1* positive neurons) signatures by calculating the top differentially expressed genes in these 2 populations using a published dataset (52) (Figure 3M). We then scored grafted neurons against these 2 signatures. We show that grafted cells classified either as A9 or A10 mDA neurons are enriched for adult SOX6+ and CALB1+ mDA signatures, respectively (Figure 3, N and O), confirming alignment with their endogenous adult counterparts. Notably, the Boost+ condition yields significantly more A9 mDA neurons across both 1-month and 9-month timepoints (Figure 3N), while the Boost protocol retains a higher authenticity score for A10 mDA neurons (Figure 3O). Moreover, the authenticity of the A9 signature increases between 1 and 9 months, suggesting continued maturation over time (Figure 3N). Collectively, these findings demonstrate robustness and long-term fidelity of the Boost+ protocol in promoting A9 mDA neuron fate, highlighting its potential for clinical translation.

### In vivo functional characterization in 6-OHDA-induced Parkinsonian rats.

**Figure 4:**
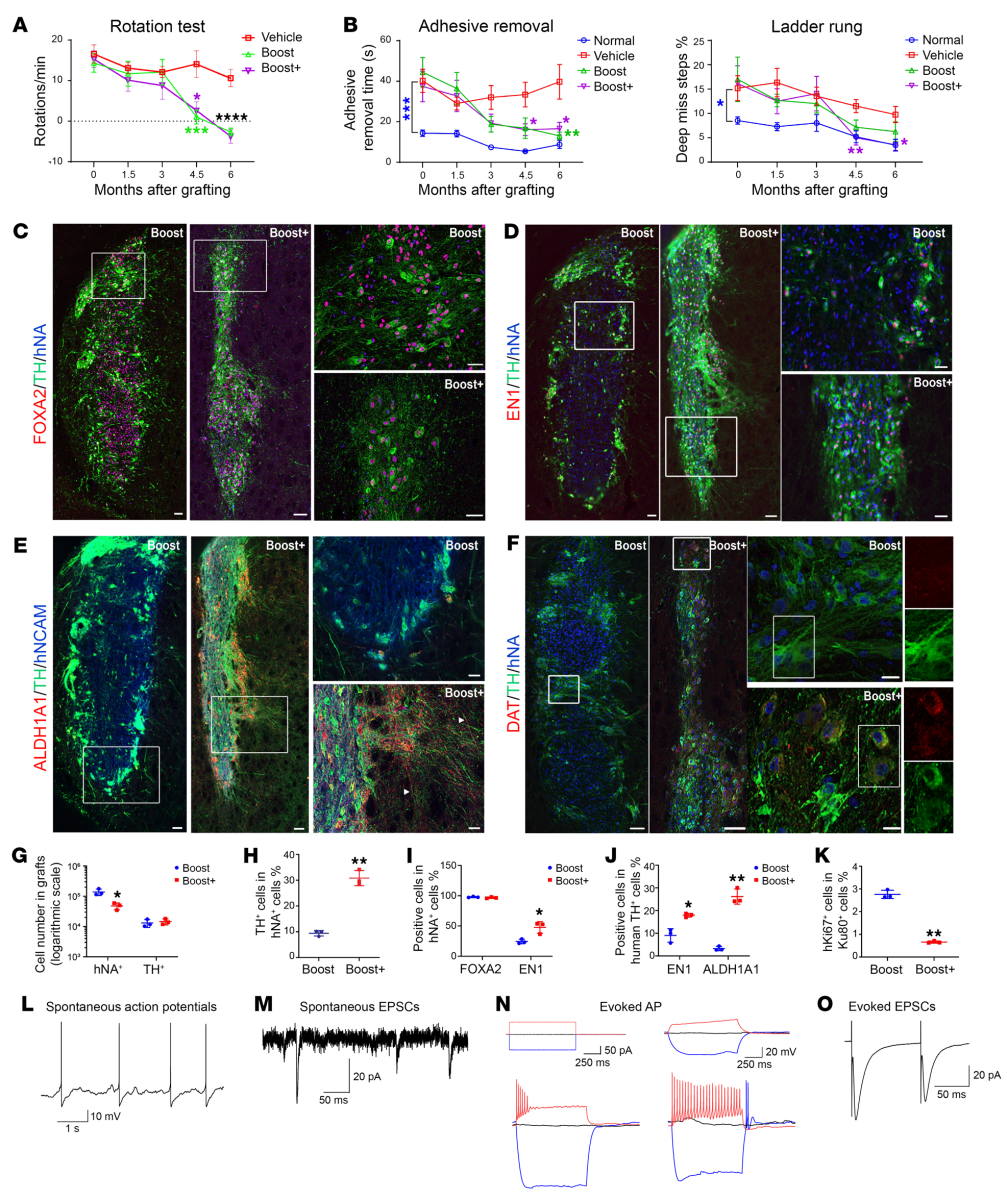
In vivo functional characterization in unilateral 6-OHDA-induced Parkinsonian rats. (**A**) Amphetamine-induced rotations per minute measured at the indicated time points after transplantation in different animal groups. (**B**) The percentage of the adhesive removal time by the contralateral paw (left) and deep miss steps in the ladder rung walking test (right). (**C**–**F**) Representative IHC images of grafted cells showing expression of FOXA2, EN1, ALDH1A1, DAT, and TH together with human-specific markers (hNA or hNCAM) in Boost and Boost+ grafts. Arrowheads indicate ALDH1A1-expressing TH^+^ dopaminergic axons (**E**). (**G**) Stereological estimation of total hNA^+^ and TH^+^ cell numbers in Boost and Boost+ grafts (logarithmic scale). (**H**–**K**) The percentage of graft composition showing TH+ cells out of hNA+ cells (**H**), FOXA2- or EN1-expressing cells out of hNA+ cells (**I**), EN1+TH+ and ALDH1A1+TH+ cells out of the total human TH+ cells (**J**), and human-specific hKi67+ cells out of human-specific hKu80+ cells (**K**) in Boost and Boost+ cell grafts. Animal numbers are 5–7 per group in **A** and **B** and 3 per group in **G**–**K**. Data are represented as mean ± SEM. *P < 0.05, **P < 0.01, ***P < 0.001. In **A** and **B**, the color of * represents the significance between the group with the same color of symbol and the vehicle group. In **C**–**E**, scale bar: 100 μm (left panels), 50 μm (right panels). In **F**, scale bar: 20 μm (right panels). The areas outlined in white lines are shown with higher magnification images in the right panels. (**L**–**O**) Whole-cell recordings from grafted mDA neurons (Boost+: 6 months after transplantation), including examples of a spontaneously active neuron (**L**), spontaneous EPSCs (**M**), action potentials evoked by current injections (**N**), and EPSCs evoked by local electrical stimulation (**O**). Recordings were performed in the absence of synaptic blockers.

Next, we grafted cryopreserved day 16 mDA precursors from Boost or Boost+ protocols into 6-hydroxydopamine-lesioned (6-OHDA-lesioned) immunodeficient rats (NIH-*Foxn1^rnu^* strain) to evaluate graft survival and impact on behavioral deficits. 6-OHDA was unilaterally injected in the median forebrain bundle with subsequent amphetamine-induced rotation tests at 4 and 6 weeks (± 1 week). Rats showing more than 6 rotations per minute were defined as completely lesioned and selected for grafting experiment. The lesioned rats were randomized into 3 groups: vehicle, Boost cell, and Boost+ groups. The Boost and Boost+ differentiations were performed on the same batch and were frozen on day 16 of differentiation. Cells were thawed and suspended at 100,000 ± 10,000 cells/μL with a viability greater than 85%. Each animal received an injection of 400,000 cells into the striatum. Animals underwent a battery of behavioral tests at 0, 1.5, 3, 4.5, and 6 months (± 1 week) after grafting, including amphetamine-induced rotation, ladder rung walking test, and adhesive removal test. Both Boost and Boost+ cell grafts led to complete recovery at 4.5 months in the rotation test (Figure 4A). At 6 months after transplantation, the average rotations per minute in the Boost and Boost+ group reached –2.68 and –3.75, respectively, compared with 10.65 in the vehicle group. In the adhesive removal test, both Boost and Boost+ cell groups showed significant recovery at 6 months after grafting (Figure 4B, left). Interestingly, only the Boost+ cell group showed significant recovery in the ladder rung walking test, compared with the vehicle group (Figure 4B, right).

Histological analysis showed that both Boost and Boost+ grafts survived and differentiated into TH^+^ mDA neurons, and extended TH^+^ processes into the surrounding host tissue (Figure 4, C–F, and Supplemental Figure 5A). Stereological cell counts showed a significantly higher number of human cells (hNA^+^) in Boost grafts (Figure 4G). The estimated graft volume was also higher in Boost (3.16 ± 0.90 mm^3^) than Boost+ (1.12 ± 0.41 mm^3^) group. This difference may be explained by the higher percentage of cycling cells at day 16 in vitro (Supplemental Figure 2E). In contrast, the overall number of TH^+^ cells was similar in both groups (Figure 4G). Accordingly, the proportion of TH+ mDA neurons was significantly higher in the Boost+ graft (30.8% versus 9.4%) (Figure 4H). The density of TH expression was also higher in the Boost+ grafts (Figure 4, C–F, and Supplemental Figure 5A). Most Boost (average at 97.7%) and Boost+ (average at 96.7%) human cells expressed FOXA2 (Figure 4, C and I), but the percentage of EN1^+^ cells was higher in Boost+ grafts, (average at 47.6% versus 27.4%) (Figure 4, D and I). The percentage of EN1^+^ in hNA^+^ TH^+^ mDA neurons is also higher in Boost+ (average of 17.9% versus 9.0%) (Figure 4, D and J). GIRK2^+^ mDA neuron percentages were higher in Boost+ (92.9 ± 1.7%) than Boost (59.7 ± 6.8%) grafts (Supplemental Figure 5B). In both Boost and Boost+ grafts, around 30% GIRK2 mDA neurons coexpressed CALB1 (Supplemental Figure 5B). Expression of ALDH1A1 was also elevated in the Boost+ grafts (average at 26% of hNCAM^+^/TH^+^ mDA neurons) compared with Boost (average at 3.3%), and extended axons into the surrounding host tissue (Figure 4, E and J). The majority (around 93%) of ALDH1A1^+^ mDA neurons coexpress GIRK2 (Supplemental Figure 5C). Dopamine transporter (DAT) expression was more pronounced in Boost+ grafts with a higher percentage of TH+ neurons expressing DAT (36.5 ± 2.4% versus 23.8 ± 0.3%) (*P* = 0.011) (Figure 4F). Assessment of proliferation (Supplemental Figure 5D) revealed a lower proportion of human Ki67^+^ cells in Boost+ grafts (average at 0.7% versus 2.8%) (Figure 4K), which may have contributed to the lower total human cell number in Boost+ grafts(Figure 4G). Nonneuronal cell types, including perivascular fibroblasts and choroid plexus-like epithelial cells, were previously reported in hPSC-derived mDA cell grafts (8, 23) but were negative in our previously reported grafts (6, 10). hCOL1A1-labeled human perivascular fibroblasts were negative in Boost grafts but present in Boost+ (Supplemental Figure 5E), with partial coexpression of PDGFR-α (Supplemental Figure 5F). Transthyretin-expressing cells were largely absent in Boost and Boost+ grafts (Supplemental Figure 5G).

Finally, we performed whole-cell recordings from graft-derived mDA neurons in the Boost+ group at 6 months after implantation. Grafted neurons exhibit spontaneous activity, spontaneous EPSCs, action potentials evoked by current injections, and EPSCs evoked by local electrical stimulation (Figure 4, L–O). In addition, whole-cell recordings from striatal medium spiny projection neurons (SPN) proximal to the mDA neuron grafts indicate that both the Boost and Boost+ grafts mediate action potential frequencies back to levels recorded in nonlesioned mice (sham). In contrast, a lower AP frequency in SPN was observed when recorded in lesioned control mice (no grafts). However, only Boost+ graft triggered a reduction in resting membrane potential (RMP) and EPSC amplitude in SPNs upon current injection conditions (Supplemental Figure 5, H–O).

## Discussion

In this study, we present the Boost+ protocol that builds on the previously published Boost method but includes FGF18 and IWP2 during neurogenic conversion. Boost+ enhances midbrain patterning and mDA neurogenesis, sustaining EN1 expression and promoting A9 and mature mDA markers, including *ALDH1A1*, *PITX3*, and *DAT*. Boost+-derived mDA neurons more closely match the transcriptional profile of in vivo human mDA neurons, and functional assays by MEA, HPLC, and electrophysiology demonstrate increased functionality. The Boost+ protocol optimizes the yield of EN1/FOXA2/LMX1A/OTX2 quadruple-positive precursors, which, in turn, promotes enhanced mDA neuron yields in vitro and in vivo. Similarly, previous work demonstrated that increased EN1 expression in mDA neuron progenitors from lineage-restricted hPSCs (4× knockout of GBX2, CDX1/2/4) (53) leads to increased TH-positive mDA neurons and improved motor recovery in a rat PD model. Additionally, we demonstrate enrichment of mDA neurons in our grafts by snNuc-Seq, while previous in vivo single-cell studies often showed grafts dominated by non-mDA neuronal populations (23, 24, 27). Cross-comparison with adult midbrain signatures confirms A9 and A10 identities over time, with Boost+ maintaining superior fidelity for A9 fate. Interestingly, WNT-pathway–associated genes were significantly enriched within prefrontal cortex–projecting A10 neurons, and computational modeling predicted that knockout of the WNT mediator TCF7L2 biases differentiation toward dorsal striatum–projecting (dSTR-projecting) A9 DA neurons (50). These findings support our observations that Boost+ enriches for A9 ALDH1A1+ mDA neurons in the graft, though how FGF18 or IWP2 may contribute to A9 lineage remains unclear.

Both protocols yielded more ALDH1A1^+^ neurons in 1-month grafts than in matched day 40 cultures, indicating that in vitro conditions were less permissive for ALDH1A1 induction or maintenance. Consistently, in vitro cells expressed lower levels of the dopamine transporters *SLC18A2* and *SLC6A3* (DAT), reflecting an immature mDA state. Since ALDH1A1+ A9 mDA neurons are highly vulnerable in PD (1), improving their in vitro specification and maintenance will be critical for future disease modeling efforts. Our data indicate greater authenticity and maturity of in vivo grafted versus in vitro cultured mDA neurons. KEGG analysis suggests enhanced Rap1 and GnRH signaling in grafted mDA neurons (Supplemental Figure 6), warranting future investigation into their roles in maintenance and maturation of ALDH1A1+ A9 mDA neurons in culture. However, it will be critical to expand on the snRNA-seq studies by testing graft composition from additional replicate differentiations and on mDA neuron grafts from independent hPSC lines.

Behavioral data show that Boost and Boost+ mDA neurons reverse drug-induced rotations and improve performance in adhesive strip removal. However, only Boost+ improves the ladder rung walking test, which is more sensitive to subtle impairment in locomotor function (54, 55), suggesting a more complete recovery of locomotion. Boost+ may present a scalable, off-the-shelf mDA cell product for clinical translation, enriched for TH+ and ALDH1A1+ A9 mDA neurons and DAT expression. However, IND-enabling studies will be required to confirm improved TH+ cell yield, to assess the reproducibility, and to track potential off-target populations. Overall cell survival remained low in Boost+-grafted mDA neurons. We recently reported the feasibility of grafting postmitotic mDA neurons and showed increased survival upon pretreatment of grafted neurons with adalimumab, an FDA-approved TNF-α inhibitor (39, 56). Combining Boost+ with cell purification and adalimumab could represent a strategy to achieve fully postmitotic mDA neuron grafts enriched for A9 subtype for preclinical studies and potential future human translation.

## Methods

### Sex as a biological variable.

Our study used both male and female hPSC lines, as detailed below. For transplantation studies, we exclusively grafted female rats, as their body weight plateaus at a manageable size compatible with standard behavioral testing apparatuses and facilitating animal handling. Past studies comparing grafting into male and female hosts did not show significant differences in outcome (10).

### hPSC culture.

Human PSCs [WA09 (H9; 46XX, WiCell), MEL1 (46XY, University of Queensland), J1 (MRC5-derived hiPSC, MSKCC)] were cultured on Vitronectin (VTN-N, Thermo Fisher #A14700) coated dishes with Essential 8 media (Life Technologies #A1517001). Passage 35–55 hPSCs were used for the experiments. hPSCs were subcultured every 4–5 days by EDTA. All cell lines are cultured at 37°C with 5% CO_2_ and routinely tested for mycoplasma.

### Transfection of hPSC.

hESCs were dissociated to single cells with Accutase (Innovative Cell Technologies # AT104) and plated at 250,000 cells/well in a vitronectin-coated 6-well plate in E8 supplemented with 10 μM Y-27632 ROCK inhibitor (Bio-Techne #1254/50). The following day, the media was replaced with mTeSR+CloneR (STEMCELL Technologies). Lipofectamine-DNA complexes (8 μL of Lipofectamine, 5 μg plasmid DNA per well) were assembled in Opti-MEM (Thermo #31985062) per manufacturer’s protocol. At 48 hours after transfection, hESCs were dissociated into single cells with Accutase, and GFP+ cells that received pX458 vector were isolated via FACS with a BD Aria6. Sorted cells were replated on vitronectin in E8 with CloneR. CloneR was withdrawn after 4 days, and clones were picked and assessed for deletion or transgene incorporation.

### Generation of reporter lines.

*GPI* targeting constructs were generated from a genomic DNA PCR with Q5 polymerase (New England Biolabs #M0494) amplifying ~500bp of homology per side and assembled with NEBuilder (New England Biolabs #E2621S). An sgRNA targeting *GPI* (GPI sgRNA: CTTCATCAAGCAGCAGCGCG) was co-transfected with its respective targeting constructs for line generation. hESC lines were transfected as described above. For reporter lines, a 1:5 ratio of sgRNA vector to targeting vector was used. Clones were screened via genomic PCR for the expected insertion.

### Directed mDA neuron differentiation.

hPSCs were dissociated into single cells using Accutase, and plated at 400K cells/cm^2^ onto Geltrex (Life Technologies, #A1413201) coated dishes with Neurobasal/N2/B27 media containing 2 mM L-glutamine, 500 ng/ml SHH C25II (R&D #464-SH), 250 nM LDN193189 (Stemgent #04-0074-02), 10 μM SB431542 (R&D systems #1614), 1μM CHIR99021 (R&D #4432), and 10 μM Rock inhibitor, which represents day 0 of differentiation, and cultured until day 3 without Rock inhibitor from day 1. From day 4 to day 6, cells were exposed to 6 μM CHIR. On day 7, LDN, SB, and SHH were withdrawn. On day 10, medium was changed to Neurobasal/B27/L-Glu supplemented with BDNF (20 ng/mL; R&D #248-BD), ascorbic acid (0.2 mM, Sigma #4034), GDNF (20 ng/mL; Peprotech #450-10), TGFβ3 (1 ng/mL; R&D #243-B3), dibutyryl cAMP (0.2 mM; Sigma #4043), and CHIR 3 μM. On day 11, cells were dissociated using Accutase and replated at 800K cells/cm^2^ on polyornithine (PO; 15 μg/mL)/ laminin (1 μg/mL)/fibronectin (2 μg/mL) coated dishes in mDA differentiation media (NB/B27/L-Glu, BDNF, ascorbic acid, GDNF, dbcAMP, and TGFβ3) until day 16. For Boost+, IWP2 (1 μM, Tocris Bioscience #3533) and FGF18 (100 ng/mL, Peprotech #100-28) were added from day 12–16. On day 16, cells were dissociated and plated using same procedure as day 11 and cultured until day 25 using mDA differentiation media+DAPT (10 μM, R&D #2634). On day 25, cells were dissociated using Accutase and replated at 200–300K cells/cm^2^) in mDA differentiation media+DAPT until endpoint. For the cryopreservation, day 16 mDA precursors dissociated with Accutase, washed, detached, made into single cells, pelleted and resuspended at 8 million cells/mL of STEM-CELLBANKER. A controlled-rate freezer (ThermoFisher) was used for cryopreservation.

### Immunohistochemistry.

Cells were fixed in 4% paraformaldehyde (PFA; Affymetrix #MFCD00133991) in DPBS for 15 min at room temperature (RT) and washed with DPBS. Cells were permeabilized with 0.5% Triton X-100 for 20 min and blocked with 2% BSA in DPBS for 30 min. Samples were incubated with primary antibodies overnight at 4°C. After washing with DPBS, samples were incubated with secondary antibodies conjugated with Alexa Fluor 488- 555-, or 647- (Thermo Fisher) at 1:400 in 2% BSA (DPBS) for 1 hour at RT in shaking incubator. Samples were counterstained with DAPI and imaged by fluorescence microscope. Primary antibodies are listed in Supplemental Table 1.

### RNA extraction and Real-time qRT-PCR.

Total RNAs were isolated with TRIzol (QIAGEN) using the Direct-zol RNA MiniPrep kit (Zymo Research, #R2052). 1 μg of RNA was used to generate cDNA using the iScript Reverse Transcription Supermix (BioRad, #170-8841). Real-time qRT-PCR was performed using the SSoFAST EvaGreen Mix (BioRad) in a BioRad CFX96 Thermal Cycler. All reactions were performed according to the manufacturer’s protocol. Results were normalized to GAPDH. Primer sequences are listed in Supplemental Table 1.

### Multi-electrode array recording.

A 100-μL droplet of medium containing 300,000 hPSC-derived mDA neurons was seeded onto poly-l-lysine-coated complementary metal oxide semiconductor multi-electrode array probes (CMOS-MEA). 1.5 mL of medium was added after 1h of incubation and replaced every 3 days. MEA recordings were performed 24 h after medium change. 1 minute of spontaneous activity was sampled from 4096 electrodes using the BioCAM system and analyzed using BrainWave 4 software (3Brain AG, Switzerland). Spike detection was performed using Timing Spike Detection (PTSD) algorithm with a detection threshold of 9 standard deviations.

### Single nuclei preparation.

Nuclei isolation protocol was adopted from previous study (57). The striatum was grossly dissected, minced on ice into small chunks, and transferred using low-attachment p-1,000 pipet by resuspending them in 1 mL of ice-cold homogenization buffer (HM). Tissue resuspension in a glass dounce homogenizer was achieved with 10 strokes of the loose (A) pestle, followed by 20 strokes of the tight (B) pestle. The homogenized suspension was transferred to a pre-chilled low DNA-bind Eppendorf tube and centrifuged for 1,000*g* for 8 minutes at 4°C. The pellets were gently resuspended in 250 μL HM buffer and subsequently mixed with 250 μL of 50% iodixanol mixture. For density gradient purification, 500 μL of the nuclei suspension was layered over 500 μL of 29% iodixanol solution and centrifuged at 13,500*g* for 20 minutes at 4°C. The nuclei pellet was resuspended in nuclei storage buffer (NSB), counterstained with DAPI, and processed for FACS to enrich human nuclei. HM, NIM buffers, iodixanol solutions, and NSB were prepared as described (57), with actinomycin D (5 μg/mL), RNasin (40 U/μL), and Superasin (20 U/μL) added to both HM and NSB.

### Single-molecule RNA fluorescent in situ hybridization (smFISH).

ViewRNA Plus ViewRNA Cell Plus Assay Kit (Invitrogen) was used under RNase-free conditions throughout experiments. Adherent cells plated in a confocal-friendly plastic bottom 24 Well Black (Ibidi) plates were fixed and permeabilized for 15 min at RT with Fixation/Permeabilization solution and blocked for 20 min followed by incubation with primary TH antibody, followed by secondary Alexa Fluor 647 antibody (Invitrogen) for 1 h at RT to locate RNA puncta signals within a mature DA neuron. Following protein detection, fluorescent in situ hybridization (FISH) and branched DNA amplification technology were used to amplify the signal detection of an RNA transcript. Z-stack images spanning the full cellular volume were acquired at 0.4-μm intervals using a Leica SP8 point-scanning confocal microscope equipped with a 63× oil-immersion objective and 3× optical zoom. Z-stacks were projected and analyzed using Imaris software to quantify RNA puncta within TH^+^ dopaminergic neurons. Eight fields of view per condition were analyzed across four independent differentiation batches. Gene targeting probes included human-specific *PITX3* and *NR4A2*, designed by ThermoFisher. PITX3-Alexa 647 Type 6 (VA6-3168220-VCP) and NR4A2-Alexa 488 (VA4-3082508-VCP) were used in the experiment.

### Intracellular protein staining and flow cytometry analysis.

Cells were dissociated into single-cell suspensions using Accutase for 30 minutes at 37°C, washed with DMEM base medium (Thermo Fisher Scientific), and filtered via 30-μm cell strainer. Cells were fixed and permeabilized using BD Cytofix/Cytoperm solution for 30 minutes at 4°C, followed by three washes with BD Perm/Wash buffer (BD Biosciences, 554723). Cells were pelleted at 500*g* for 5 minutes at 4°C, resuspended, and incubated with primary antibodies diluted in Perm/Wash buffer for 30 minutes at 4°C. Primary antibodies against FOXA2, OTX2 (R&D Systems), and EN1 (Invitrogen) were used. Following primary antibody incubation, cells were washed and incubated with fluorophore-conjugated secondary antibodies (1:5,000) for 30 minutes at 4°C. After washing, cells were analyzed using a FACSAria III flow cytometer (BD Biosciences). Isotype and secondary-only controls were included to establish gating strategies.

### Single-cell analysis.

Samples were processed with 10x Chromium 3′ v3, aligned to GRCh38 (and mm10 for grafts) using Cell Ranger v5.0.0, filtered for human cells (grafts), and analyzed in Scanpy v1.9.3 after removing genes detected in fewer than five cells.

In vitro and graft datasets were processed separately with dataset-specific QC. In vitro cells with <500 or >2000 genes and >10% mitochondrial RNA were excluded. Graft cells were retained if they had 1000–5000 genes and <0.25% (1 month) or <1% (9 months) mitochondrial RNA to limit artifacts, doublets, and stressed cells. Highly variable genes were identified using default Scanpy settings, with regression of total counts and mitochondrial content. Data were normalized and log-transformed, and KNN graphs were built using 50 PCs and 10 neighbors for in vitro data, and 20 PCs with 20 neighbors for 1- and 9-month graft datasets. Batch effects were corrected using BBKNNClick or tap here to enter text. Cell communities were identified using the Leiden algorithm (resolution 1.0 in vitro; 0.9 at 1 month and 0.8 at 9 months in grafts), visualized by UMAP, and annotated using canonical markers. Cell-type connectivity was assessed using PAGA ^16^ in Scanpy, excluding unknown cells and retaining edges with weights >0.2. For *TH^+^* or *NR4A2^+^* subsets, data were processed with scran v1.22.1, selecting 2000 highly variable genes, computing 50 principal components, and constructing shared nearest neighbor graphs using buildSNNGraph.

Clusters were identified using the walktrap algorithm (cluster_walktrap, igraph v1.3.5) and visualized by UMAP. Differential expression was performed with Seurat, and pathway enrichment with clusterProfiler v4.2.2. Marker-positive cells were defined using gene-specific thresholds (median + 1 SD). Differences in marker co-expression between protocols were assessed by two-sided permutation tests (10,000 permutations), with p-values derived from the null distribution, and 95% confidence intervals estimated by bootstrap resampling (10,000 iterations).

### Similarity score.

To assess enrichment toward authentic midbrain DA neurons, we compared Boost and Boost+ cells to human fetal (in vitro and graft) and adult (graft only) midbrain DA single-cell references. We identified A9 (*SOX6^+^*) and A10 (*CALB1^+^*) signatures by computing the top differentially expressed genes (Wilcoxon rank-sum; Benjamini–Hochberg correction) from published fetal (49) and adult (52) datasets. The top 100 genes per signature were used to calculate per-cell similarity scores (mean expression of signature genes minus a random-gene baseline). Scores were visualized by protocol and compared across protocols and time points using the Mann–Whitney test with Benjamini–Hochberg correction.

### Animals.

Athymic nude rats (NIH-Foxn1rnu, 6-8 weeks old, female, Charles Rivers Laboratory) and NSG mice (NOD.Cg-Prkdcscid Il2rgtm1Wjl/SzJ, 6-8 weeks old, male, Jackson Laboratory) were included in the studies.

### 6-OHDA lesioning and cell grafting.

The animals were anesthetized by Isoflurane during the surgeries. For athymic nude rats, to establish unilateral medial forebrain bundle lesions of the nigro-striatal pathway, 6-OHDA solution (3.6 mg/mL in 0.2% ascorbic acid and 0.9% saline, Millipore) was stereotactically injected to (2.5 μL, Tooth bar set at −2.4, AP −4.4 mm, ML −1.2, VL −7.8; 3 μL, Tooth bar set at +3.4, AP −4.0 mm, ML −0.8, VL −8.0). Human PSC-derived day 16 mDA progenitors were resuspended at 100,000 ± 10,000 cells per microliter in transplantation medium consisting of neurobasal medium with 200 mM L-glutamine and 100 mM ascorbic acid (AA), 0.1% Kedbumin. The cell suspension was delivered to 4 deposits (1 μL per deposit) into the rat striatum (AP: +1 mm, ML: −2.8, VL: −4.7, −4.6, −4.5 and −4.4 mm from dura) or 2 deposits along the DV axis (1 μL per deposit) into the mouse striatum ([AP] +0.5 mm, [ML] +/–1.8 mm, [DV] –3.4 to –3.3 mm from dura) at the rate of 0.5–1 μL/min via a motorized stereotaxic injector (Model 53311, Stoelting company, IL, USA). The syringe was kept in place for 5 minutes, then withdrawn at 1 mm/min. All cells used for transplantation studies underwent proper quality control (QC) metrics prior to injection such as immunofluorescence, intracellular flow, qPCR, and trypan blue or AOPI viability assays.

### Behavior tests.

Amphetamine-induced rotation, ladder rung walking test, adhesive removal task were performed before transplantation, and at 1.5, 3, 4.5, 6 months after transplantation. The animals were habituated for 30 minutes before the behavior tests. For the amphetamine-induced rotation test, the rats were injected intraperitoneally with D-Amphetamine in saline (Sigma, 5 mg/kg). The rotations were recorded for 40 minutes and automatically counted by Ethovision XT 16. The data were presented as (Ipsilateral-contralateral) rotations per minute. The ladder rung walking test was performed on the foot misplacement corridor (Panlab) with irregular rung arrangements (54). The percentage of missed steps out of total steps was calculated. The adhesive removal task was performed with the adaptation that the adhesive tape was applied onto the forepaws (58). The time the animal required to remove the tape from the left paw was recorded.

### Tissue processing, IHC, image processing and stereological analysis.

Mice were anesthetized with pentobarbital and transcranially perfused using heparinized PBS (10 U/mL, pH 7.4), followed by 4% PFA in PBS. Brains were post-fixed in ice-cold 4% PFA for 18 hours, cryoprotected in 30% sucrose, frozen in O.C.T (Fisher Scientific), and cryosectioned at 30 μm onto Superfrost plus microscope slides (Fisher Scientific). Slides were air-dried for 18 hours at RT and stored at –80°C for long-term use. For immunolabeling, tissue sections were washed twice in PBS and permeabilized with 0.5% Triton X-100 in PBS for 10 minutes. Sections were incubated with primary antibodies in 2% BSA and 0.25% Triton X-100 in PBS overnight at 4°C. The following day, samples were incubated with appropriate Alexa–conjugated secondary antibodies (1:500) for 30 minutes at RT. All antibodies are listed in Supplemental Table 1.

Rats were perfused with 4% PFA. Brain tissues were dissected and post-fixed with 4% PFA for 12 hours, then changed to 30% sucrose in 0.01 M PBS for 24 hours. The tissues were embedded in O.C.T (Sakura Finetek USA, Inc.) and cryosectioned at 30 μm. DAB staining was performed on a Leica Bond RX (MSKCC molecular cytology core) using EDTA-based epitope retrieval (ER2, 20 mins at 100°C), TH antibody (1 μg/mL, 60 min, RT), Leica Bond Polymer anti-rabbit HRP (8 min). DAB (10 min), and Hematoxylin counterstain (10 min) from the Polymer Refine Detection Kit (Leica, DS9800). After staining, sample slides were washed in water, dehydrated using ethanol gradient (70%, 90%, 100%), washed three times in HistoClear II (National Diagnostics, HS-202), and mounted in Permount (Fisher Scientific, SP15). Stained slides were scanned on a Panoramic Scanner (3DHistech) at 20x/0.8NA and analyzed by Caseviewer 2.4 software (3DHistech Ltd). Stereological estimation was performed using Stereo Investigator (MBF Bioscience) including optical fractionator probe (cell number) and Cavalieri estimation function (graft volume).

### Electrophysiological recordings in cultured mDA neurons.

Patch-clamp electrophysiological recordings were performed on randomly selected hPSC-derived mDA neurons at day 60 at RT in a Tyrode’s solution containing (in mM): 119 NaCl, 3 KCl, 10 glucose, 2 CaCl_2_, 1.2 MgCl_2_-6 H_2_O, 3.3 HEPES, and 2.7 HEPES-Na^+^ salt (pH 7.4, 270 mOsm). Whole-cell patch-clamp recordings were performed using borosilicate pipettes (3–4 MΩ) filled with K-gluconate internal solution, a MultiClamp 700B amplifier, and WinWCP software, as previously described (6). In each cell, input resistance (measured by −100 pA, 1s hyperpolarizing pulse), resting membrane potential, and spontaneous action potentials were monitored throughout the recording. Current-voltage relationship and evoked action potentials were measured by injecting a somatic current (1 s duration) from −30 to +20 pA in +10 pA increments and from 0 to +250 pA in +10 pA increments, respectively.

### Electrophysiological recordings in grafted mDA neurons.

Coronal striatal slices (250 μm thick) were prepared from adult mice using a vibratome (Leica VT1200) and ice-cold cutting solution containing (in mM): 194 sucrose, 30 NaCl, 26 NaHCO_3_, 4.5 KCl, 1 MgCl_2_-6 H_2_O, 1.2 NaH_2_PO_4_-6 H_2_O and 10 glucose. Slices were allowed to recover in the solution for 30 min at 34°C and then transferred to ACSF saline containing (in mM): 125 NaCl, 2.5 KCl, 26 NaHCO_3_, 2.4 CaCl_2_, 1.3 MgCl_2_-6 H_2_O and 0.8 NaH_2_PO_4_-6 H_2_O, and 10 glucose. Recordings were performed at 34 ± 2°C. For whole-cell current patch clamp recordings, K-gluconate pipette solution described for recording in culture was used. For a subset of voltage-clamp recordings to measure EPSCs, the pipette solution contained (in mM): 120 cesium-methanesulfonate, 11 glucose, 10 HEPES, 5 NaCl, 2 NaATP, 2 MgATP, 1.1 EGTA and 0.3 NaGTP (pH 7.3, 270–273 mOsm). In each cell, input resistance (measured by –50 pA, 1 s hyperpolarizing pulses), resting membrane potential, spontaneous action potentials and EPSCs were monitored throughout the recording. EPSCs were evoked using a concentric bipolar electrode (World Precision Instruments) placed approximately 100 μm away from recorded cells, with stimulation controlled by Master-9 (A.M.P.I) and the stimulation current intensity was controlled by Iso-flex (A.M.P.I). To examine the effect of the graft on striatal glutamatergic transmission, spiny projection neurons (SPNs) near the graft core or adjacent processes were patch clamped and their input resistance and baseline resting membrane potential were monitored in the current clamp mode. Voltage-current relationship was measured by injecting step current from −300 to + 100 pA with +50 pA increments. The excitability and the rheobase of SPNs were measured by injecting step current from 0 to +650 pA with +50 pA increments. Synaptic efficacy was measured by averaging 5 EPSCs (10 s intervals) at each stimulation intensity (0–25 μA, 100 μs duration). Recordings with >20% series change were excluded. Data were analyzed in Clampfit (Molecular Devices, CA) and were presented as mean ± SEM. Statistical analysis was performed by one-way and two-way ANOVA test with Sidak’s multiple comparisons test (GraphPad Prism 8).

### HPLC.

mDA neurons were plated on PO/laminin/fibronectin-coated 24-well plates at 5 × 10^5^ cells/well density on day 25 and used at day 60. HPLC with electrochemical detection as previously described (59). Briefly, cells were preincubated in fresh DMEM: F12 + N2 medium for 30 min. After exposure to either Tyrode’s saline alone or supplemented with high KCl (80 mM, Sigma) for 5 min at RT, supernatant was collected and immediately mixed with 0.2 M perchloric acid (1:1 volume) to deproteinize the sample and prevent dopamine auto-oxidation. Perchloric acid was also added into the wells with cells to measure intracellular DA concentration. After 10 min incubation at RT, samples were centrifuged at 10,000 g for 5 min at 4°C, supernatant was collected, stored at –80°C and analyzed within two weeks. DA concentrations in each group of samples were normalized to the levels in the corresponding control group; data were averaged from 2 independent experiments. The intracellular and extracellular DA concentrations were divided by the fraction of DA neurons in each group (0.25 in Boost and 0.41 in Boost+). Those percentages were derived from the scRNA-seq analysis.

### Statistics.

In all studies, animals were randomized into different groups. Data were represented as mean ± SD unless indicated as mean± SEM. The number of cases in groups is specified in the figure legends. One-way ANOVA was applied, and an unpaired 2-tailed Student’s *t* test was used between 2 groups unless otherwise indicated. Welch’s correction was applied to data with unequal SDs. Probability (p) values of less than 0.05 were considered statistically significant. All statistical analyses were performed using GraphPad Prism 9. To reduce the observation bias, the individuals who performed the experiments were blinded to group assignments in all procedures. Animals without any grafts due to technical failure during transplantation were not included in the graft analyses.

### Study approval.

The use of human PSCs in this experimental setting has been approved by the Tri-Institutional ESCRO committee (Tri-SCI ESCRO; New York, NY). All animal procedures were approved by our Institutional Animal Care and Use Committee (IACUC; MSKCC, New York, NY) and follow NIH guidelines.

### Data availability.

All sc/snRNA-seq data have been deposited in the ArrayExpress database at EMBL-EBI (www.ebi.ac.uk/arrayexpress/) under accession no. E-MTAB-14729. Supporting data values for all figures are provided in the Supporting Data Values file.

## Author contributions

Conceptualization, TWK, JP, VT, and LS. Writing – Original Draft, TWK, JP, VDB, and LS. Dopamine neuron differentiation and characterization, TWK, SYK, EH, RW, YEI, and HJJ. In vivo transplantation and analysis of the data, JP, SYK, LRP, SJ, ZAM, NC, and SAD. Bioinformatics Analysis and interpretation of the data, VDB, FC, DY, HSC, and DB. Electrophysiology and dopamine release experiments and analysis of the data, SJC, AKF, and EVM; Funding Acquisition, LS, VT, TWK, and DB. All authors provided feedback on editing the manuscript. Co-first authorship was determined by the independent and complementary leadership of the 3 cofirst authors in hPSC differentiation study (TWK), preclinical model development and analysis (JP), and computational analyses in vitro and in vivo (VDB).

## Conflict of interest

LS and VT are cofounders, scientific advisors, and have received research support from BlueRock Therapeutics. LS is a cofounder of DaCapo Brain Science. Memorial Sloan Kettering has filed a patent application related to the boost+ differentiation protocol with LS, TWK, and SK listed as inventors (US20230143486A1).

## Funding support

This work is the result of NIH funding, in whole or in part, and is subject to the NIH Public Access Policy. Through acceptance of this federal funding, the NIH has been given a right to make the work publicly available in PubMed Central.

- NIH grants 1R01NS118067-01A1 (to LS, DB), R01NS126588 (to VT).
- The National Research Foundation of Korea (NRF) grant from the Korea government (MSIT) (No. RS-2024-00351442; to TWK).
- The Freedom-Together-Foundation and from BlueRock Therapeutics (to LS).

## Supplementary Material

Supplemental data

Supplemental table 1

Supporting data values

## Version 1 05/15/2026

Electronic publication
