# Supplementary material for: Modulation of WNT and FGF18 enhances yield and subtype identity of hPSC-derived midbrain dopamine neurons
Source: J Clin Invest. 2026 May 15;136(10):e190954. doi: 10.1172/JCI190954 (PMC13178649; doi:10.1172/JCI190954)
Supplement: Supplemental data [file jci-136-190954-s091.pdf]

Supplemental Figure 1. FGF18 and IWP2 treatment improves EN1+FOXA2+LMX1A+OTX2 mDA cells while reducing off-target-related genes across diverse hPSCs

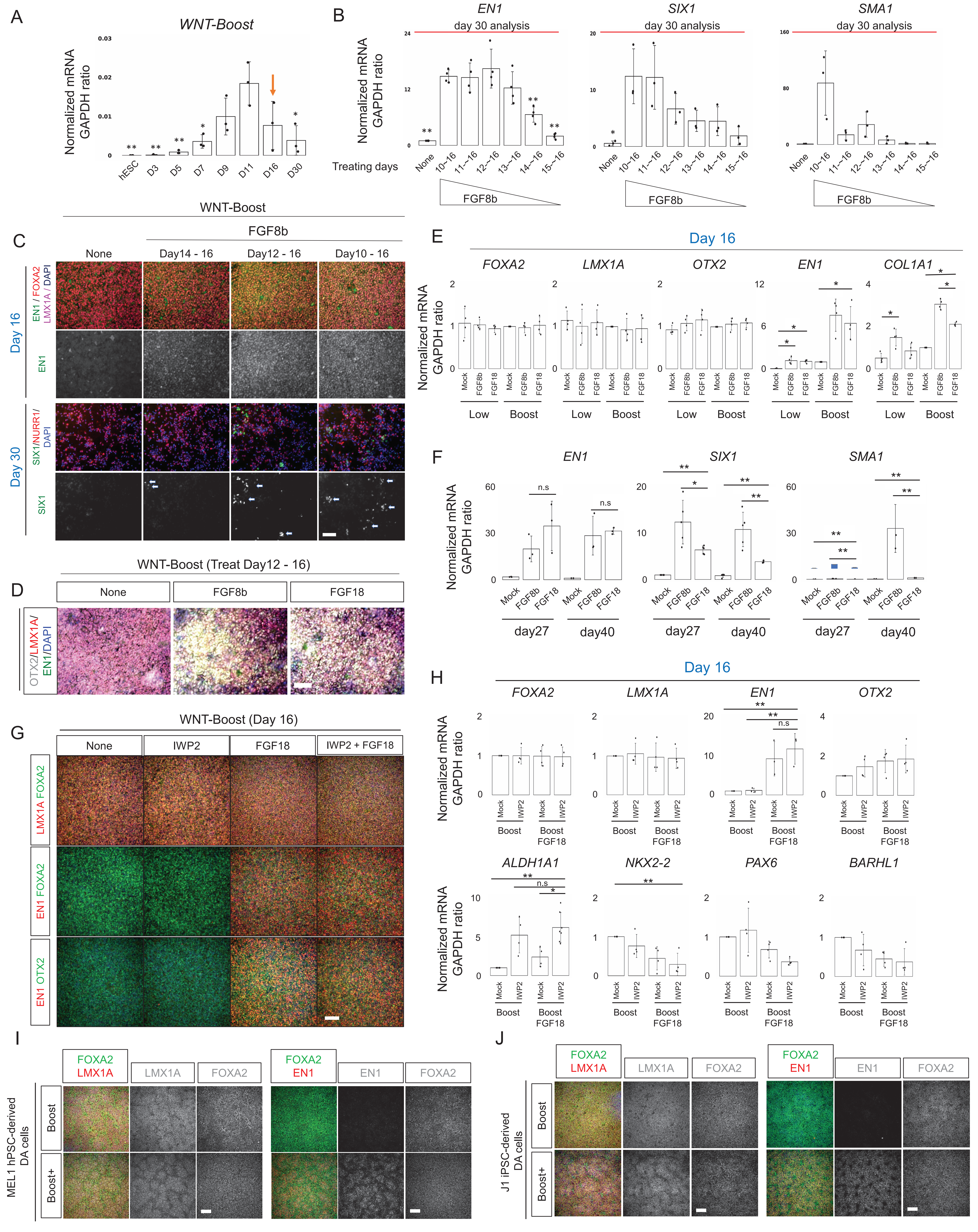

Supplemental Figure 2. scRNA-seq analysis, characterization of COL1A1 expressing cells, and cell surface markers mediated mDA neuron purification.

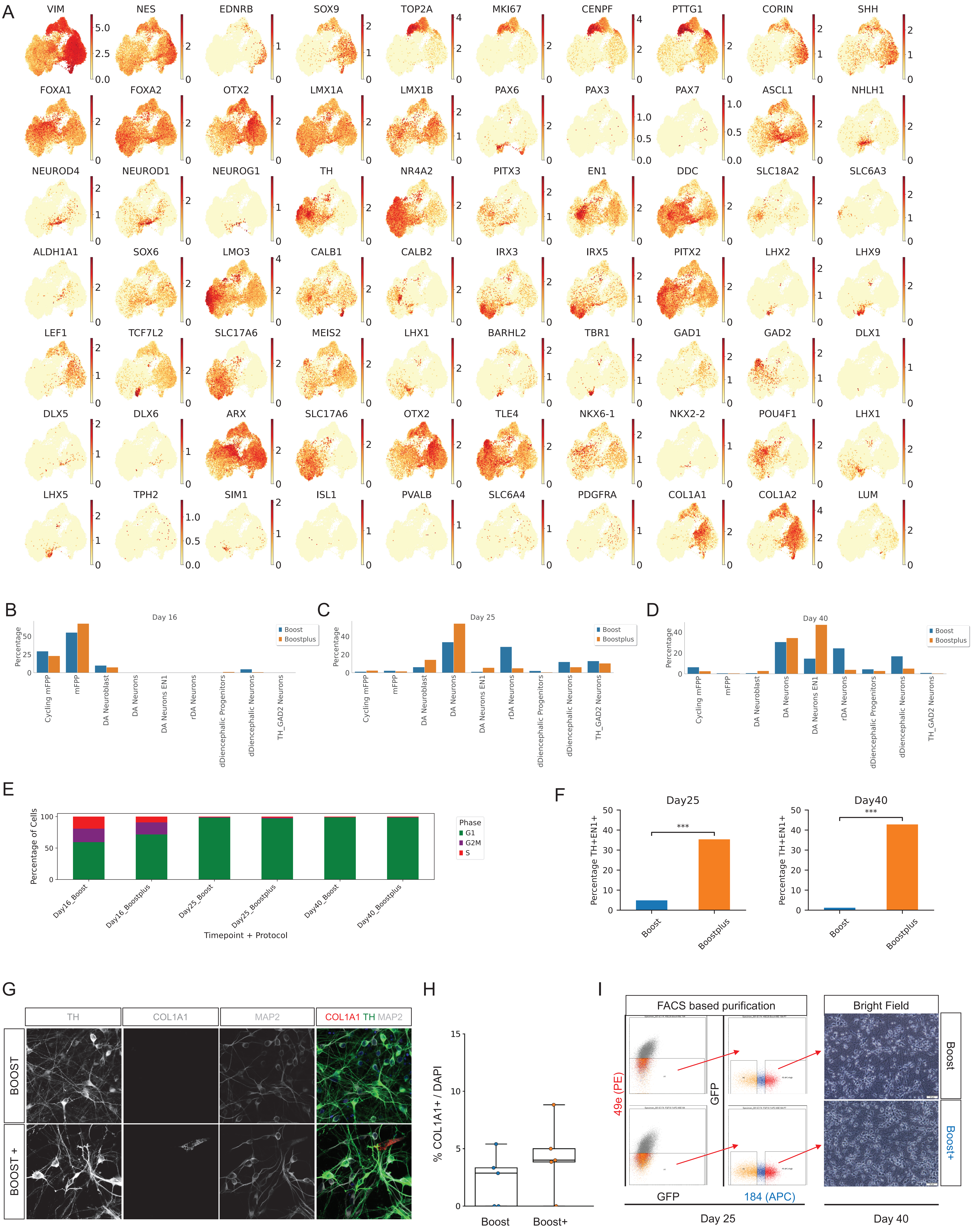

Supplemental Figure 3. Characterization of cell type composition within grafts at 1 month by snRNA-seq

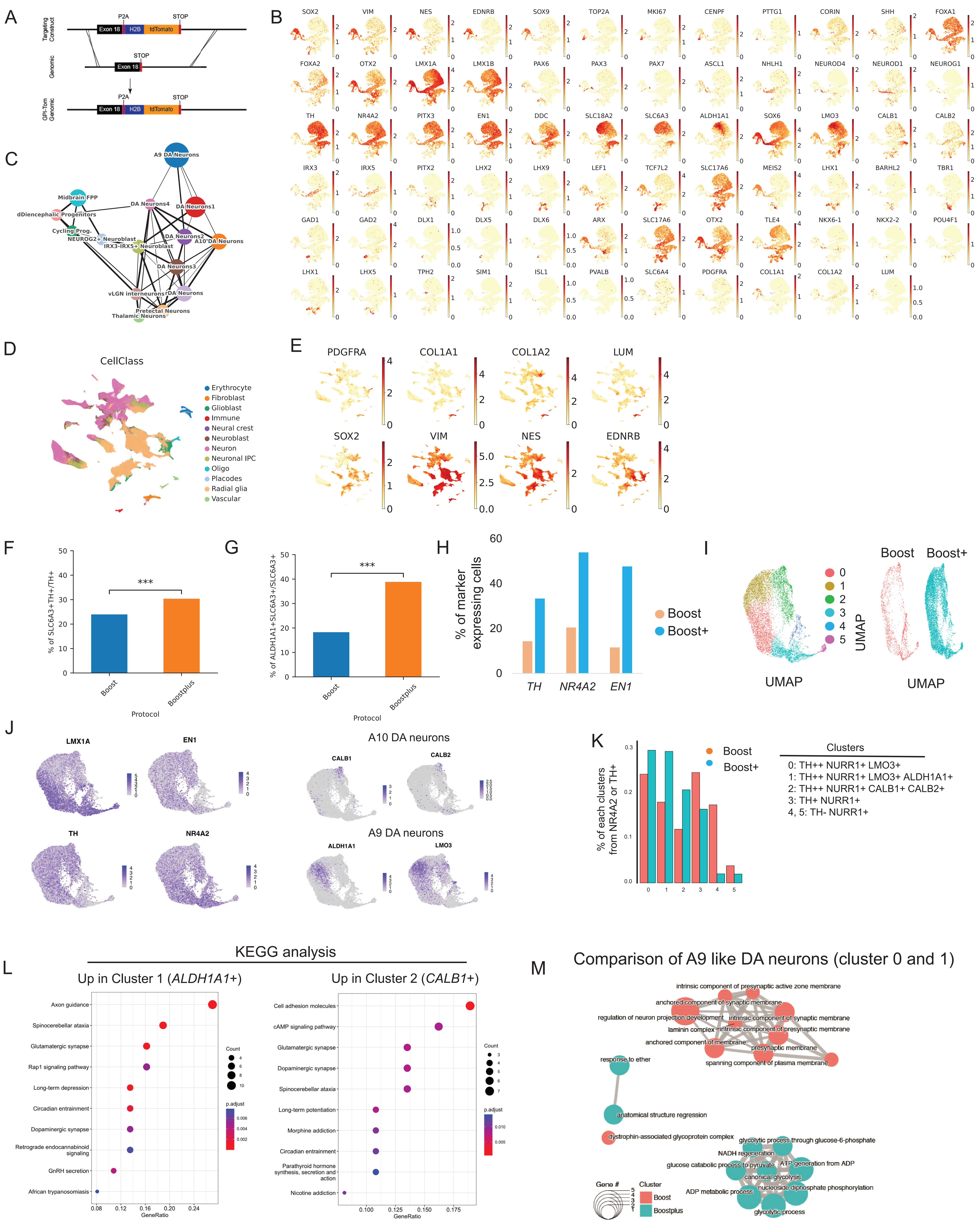

Supplemental Figure 4. Cell type composition within grafts by snRNA-seq at 1- and 9-months

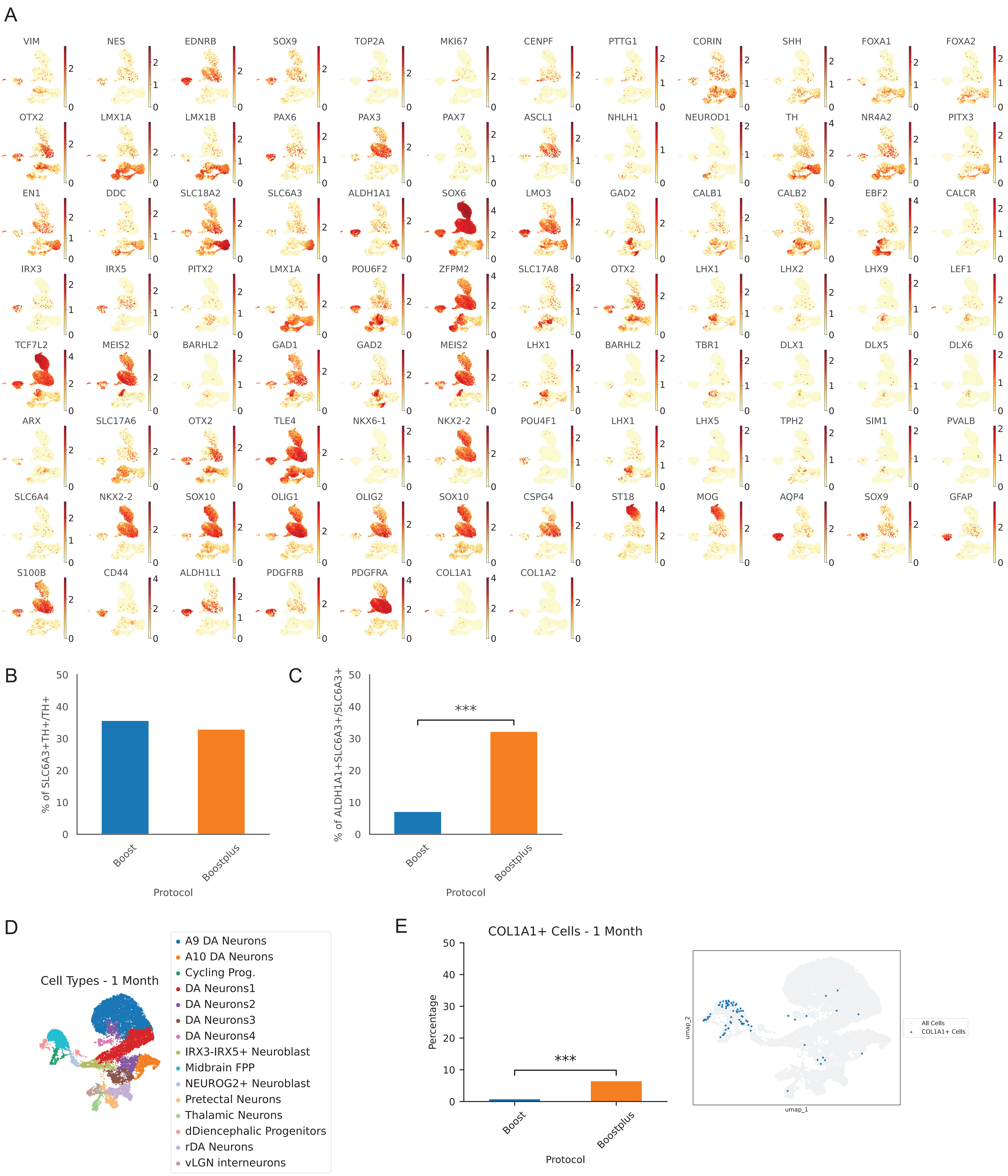

Supplemental Figure 5. Characterization of the Boost and Boost+ grafts in unilateral 6-OHDA lesioned rat striatum

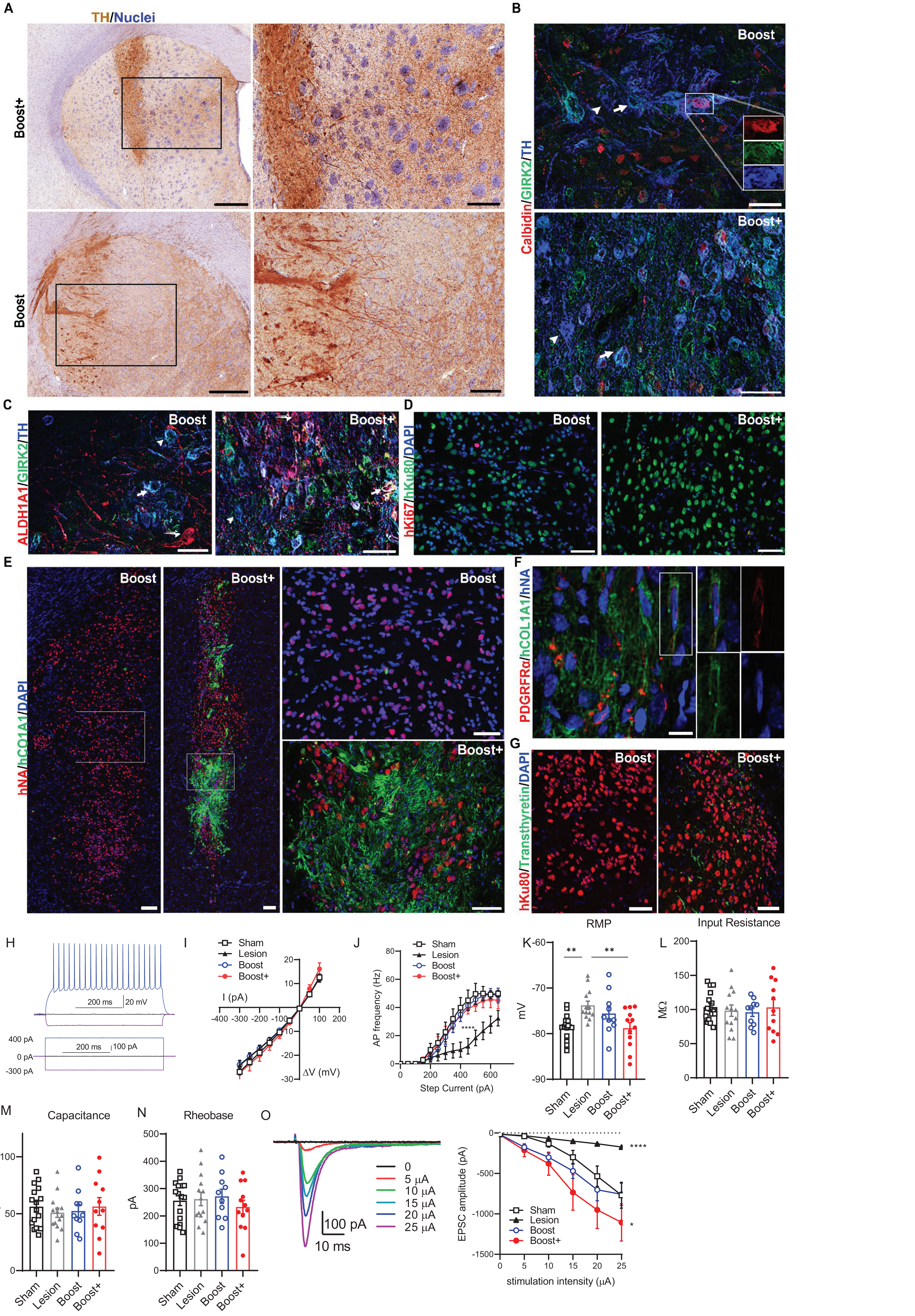

Supplemental Figure 6. KEGG-GO analysis of NURR1+ and TH+ mDA neurons comparing in vivo grafts (snRNA-seq) and age-compatible in vitro mDA neurons (scRNA-seq, Day40) in Boost and Boost+ conditions

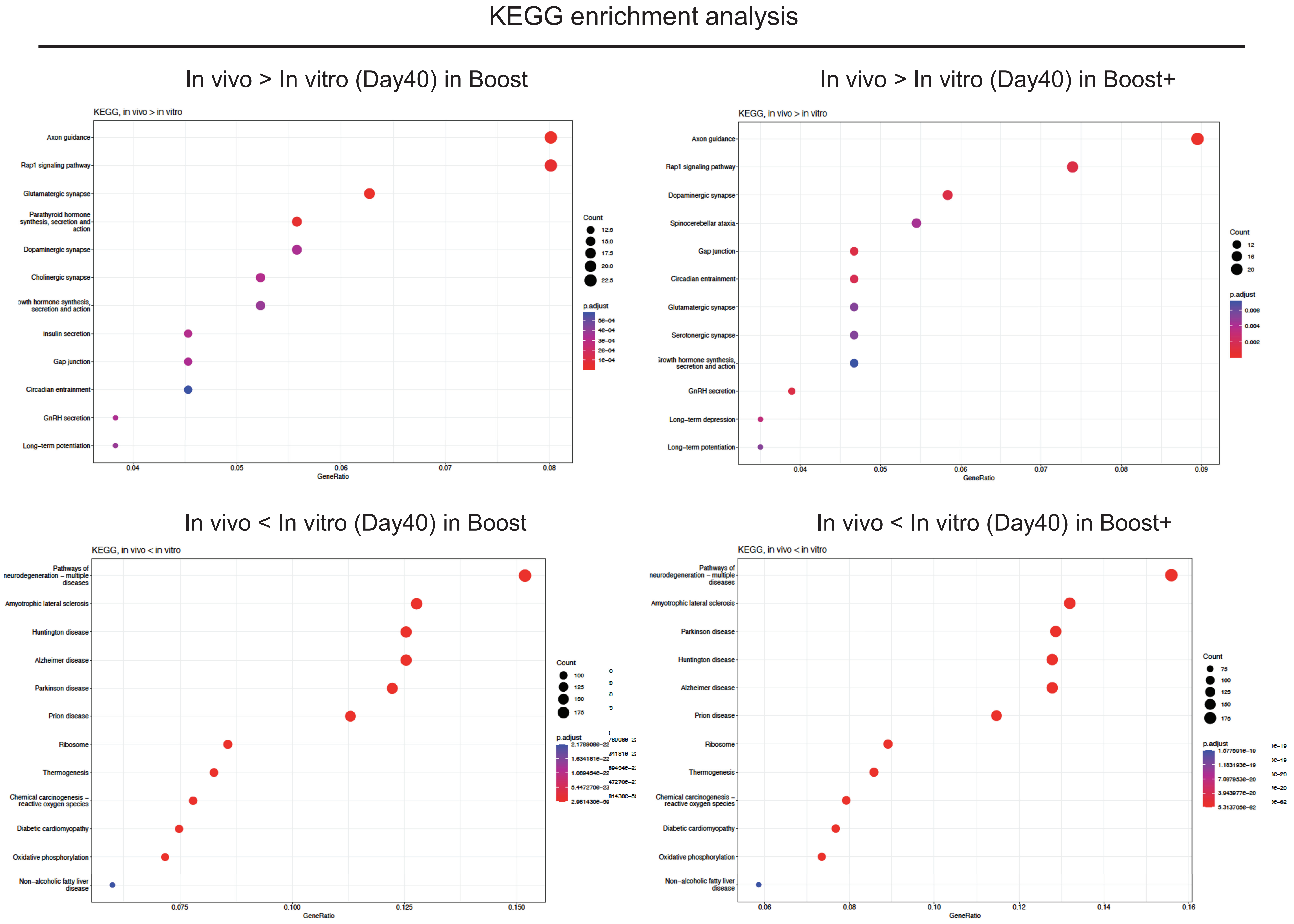

SUPPLEMENTAL FIGURE LEGEND

SUPPLEMENTAL FIGURE 1. FGF18 and IWP2 improve EN1+FOXA2+LMX1A+OTX2+ mDA cells while reducing off-target-related genes across diverse hPSCs.

(A) qRT-PCR analysis of *EN1* expression during mDA differentiation using the Boost protocol. **(B)** qRT-PCR analysis of mDA differentiated cells for *EN1* and non-mDA markers (*SIX1* and *SMA1*) at day 30 following variable durations of FGF8b treatment (day 10–15 to day 16) on the Boost protocol. **(C)** Representative immunofluorescent image of mDA differentiated cells for FOXA2, LMX1A, and EN1 at day 16, and SIX1 and NURR1 at day 30, following indicated FGF8b treatment windows on the Boost protocol. **(D)** Immunofluorescence analysis of EN1, OTX2, and LMX1A at day 16 after FGF8b or FGF18 treatment from day 12 to day 16. **(E)** qRT-PCR assay of mDA differentiated cells on day 16 with FGF8b and FGF18 from day 12 - 16 on the Low condition (without WNT-Boost step; continue 1uM CHIR until day 11 during differentiation) and the Boost protocol. **(F)** qRT-PCR assay of mDA differentiated cells on day 27 and day 40 with treatment of FGF8b and FGF18 from day 12 - 16 on the Boost protocol. **(G)** Representative immunofluorescent image of mDA differentiated cells for mDA markers at day 16 with treatment of IWP2 and/or FGF18 from day 12 - 16 on the Boost protocol. **(H)** qRT-PCR analysis of mDA differentiated cells on day 16 from (**G**). **(I, J)** Representative immunofluorescent image of mDA differentiated cells for mDA markers at day 16 derived from MEL1 hESC (**I**) and J1 iPSC (**J**) by the Boost and Boost+ protocol. Data are represented as mean ± SD. *P<0.05, ** P<0.001 (two-way Student's t-test).

SUPPLEMENTAL FIGURE 2. scRNA-seq analysis, characterization of COL1A1+ cells, and cell surface marker-mediated mDA neuron purification. (A) UMAP plot showing expression levels of markers of general neuronal progenitors (*VIM*, *NES*, *EDNRB*, *SOX9*), cycling progenitors (*TOP2A*, *MKI67*, *CENPF*, *PTTG1*), midbrain floor progenitors (*CORIN*, *SHH*, *FOXA1*, *FOXA2*, *OTX2*, *LMX1A*, *LMX1B*), dorsal diencephalic progenitors (*PAX6*, *PAX3*, *PAX7*), neuroblasts (*ASCL1*, *NHLH1*, *NEUROD4*, *NEUROD1*, *NEUROG1*), general dopamine neurons (*TH*, *NR4A2*, *PITX3*, *EN1*, *DDC*, *SLC18A2*, *SLC6A3*), A9 mDA neurons (*ALDH1A1*, *SOX6*, *LMO3*), A10 mDA neurons (*CALB1*, *CALB2*), subthalamic neurons (*IRX3*, *IRX5*, *PITX2*), thalamic neurons (*LHX2*, *LHX9*, *LEF1*, *TCF7L2*, *SLC17A6*), pretectal neurons (*MEIS2*, *LHX1*, *BARHL2*, *TBR1*), vLGN interneurons (*GAD1*, *GAD2*, *DLX1*, *DLX5*, *DLX6*, *ARX*, *SLC17A6*, *OTX2*, *TLE4*), red nucleus (*NKX6-1*, *NKX2-2*, *POU4F1*, *LHX1*, *LHX5*, *TPH*), OMTN (*SIM1*, *ISL1*, *PVALB*), serotonergic neurons (*SLC6A4*), and VLMCs (*PDGFRA*, *COL1A1*, *COL1A2*, *LUM*). **(B-D)** Percentage of cell types in each protocol at day 16 (**B**), day 25 (**C**)**,** and day 40 (**D**). **(E)** Proportion of cells in different phases of the cell cycle, stratified by day and protocol. **(F)** Percentage of cells co-expressing *EN1* and *TH* in *TH*-expressing cells from the scRNA-seq. Day 25 (Δ = +30.46%, p < 0.0001, permutation test; 95% bootstrap CI: [27.54%, 33.36%]) and at Day 40 (Δ = +41.86%, p < 0.0001; 95% bootstrap CI: [38.84%, 44.98%]). **(G-H)** Representative immunofluorescent image **(G)** of mDA differentiated cells for TH and COL1A1, and quantification **(H)** of COL1A1 positive cells from (**G**). **(I)** Flow cytometry–based purification strategy for post-mitotic mDA neurons using low CD49e and high CD184 expression (**left**), and bright-field image of purified mDA neurons, two weeks after sorting (**right**).

SUPPLEMENTAL FIGURE 3. Characterization of cell type composition within grafts at 1 month by snRNA-seq

(**A**) Schematic of the reporter strategy used to isolate human graft-derived mDA neurons expressing tdTomato for snRNA-seq analysis. **(B)** UMAP plot showing expression levels of markers of general neuronal progenitors

(SOX2,*VIM*,*NES*,*EDNRB*,*SOX9*), cycling progenitors (*TOP2A*,*MKI67*,*CENPF*,*PTTG1*), midbrain floor progenitors (*CORIN*,*SHH*,*FOXA1*,*FOXA2*,*OTX2*,*LMX1A*,*LMX1B*), dorsal diencephalic progenitors (*PAX6*,*PAX3*,*PAX7*), neuroblasts (*ASCL1*,*NHLH1*,*NEUROD4*,*NEUROD1*,*NEUROG1*), general dopamine neurons (*TH*,*NR4A2*,*PITX3*, *EN1*, *DDC*, *SLC18A2*, *SLC6A3*), A9 mDA neurons (*ALDH1A1*,*SOX6*,*LMO3*), A10 mDA neurons (*CALB1*,*CALB2*), subthalamic neurons (*IRX3*,*IRX5*,*PITX2*), thalamic neurons (*LHX2*,*LHX9*,*LEF1*,*TCF7L2*,*SLC17A6*), pretectal neurons (*MEIS2*,*LHX1*,*BARHL2*,*TBR1*), vLGN interneurons (*GAD1*,*GAD2*,*DLX1*,*DLX5*,*DLX6*,*ARX*,*SLC17A6*,*OTX2*,*TLE4*), red nucleus (*NKX6-1*,*NKX2-2*,*POU4F1*,*LHX1*,*LHX5*,*TPH*), OMTN (*SIM1*,*ISL1*,*PVALB*), serotonergic neurons (*SLC6A4*), and VLMCs (*PDGFRA*,*COL1A1*,*COL1A2*,*LUM*). **(C)** Abstracted graph showing the relationships between cell states. **(D, E)** t-SNE plot of the fetal brain single-cell atlas (49) colored by cell type (**D**) and by VLMC/fibroblast (*PDGFRA*,*COL1A1*,*COL1A2*,*LUM*), and neural progenitor markers (SOX2,*VIM*,*NES*,*EDNRB*) (**E**). **(F, G)** Percentage of *TH+SLC6A3+* cells in *TH+* cells (Δ = +6.42%, $p < 0.0001$), and *ALDH1A1+SLC6A3+* cells in S*LC6A3+* cells (Δ = +20.66%, $p < 0.0001$). **(H)** Proportion of cells expressing mDA markers (*TH*, *NR4A2*, *EN1*) in Boost and Boost+ grafts. **(I)** UMAP visualization of combined graft-derived cells(**left**) and protocol-separated clusters(**right**) from Boost and Boost+ conditions. **(J)** UMAP feature plots of selected marker genes defining neuroblasts, mDA neurons, and A9 and A10 mDA subtypes. **(K)** Percentage of each cluster from the *NR4A2*+ or *TH*+ cells by the Boost and Boost+. **(L)** KEGG-GO analysis of the upregulated pathway in high expression of *ALDH1A1* and *CALB1* clusters. **(M)** Plot showing pathways enriched in the A9 mDA cells generated using the Boost (red) or Boost+ (green) grafts.

SUPPLEMENTAL FIGURE 4. Cell type composition within grafts by snRNA-seq at 1- and 9-months

(A) UMAP plot showing expression levels of marker genes as in legend S3B with the addition of OPC/oligodendrocytes (*NKX2-2*, *SOX10*, *OLIG1*, *OLIG2*, *SOX10*, *PDGFRA*, *CSPG4*, *ST18*, *MOG*), and astrocytes markers (*AQP4*, *SOX9*, *GFAP*, *S100B*, *CD44*, *ALDH1L1*, *PDGFRB*). **(B, C)** Percentage of cells co-expressing *TH* and *SLC6A3* in *TH+* cells and co-expressing *ALDH1A1* and *SLC6A3* in *SLC6A3+* cells (Δ = +25.08%, $p < 0.0001$) at 9 months. **(D, E)** Identification and quantification of COL1A1^{+} cells within 1-month floor plate progenitors. UMAP showing annotated cell types at 1-month post-differentiation, including midbrain floor plate progenitors (Midbrain FPP) (**D**)**.** UMAP highlighting COL1A1^{+} cells (blue) within the overall population (gray), with localization predominantly within the Midbrain FPP cluster (**E, right**). Quantification of COL1A1^{+} cells (**E, left**) across protocols reveals a significantly higher proportion in the Boost+ condition compared to Boost in FPP (Δ = +5.64%, $p < 0.0001$, permutation test), suggesting early enrichment of COL1A1^{+} progenitors that may give rise to VLMCs at later timepoints in the Boost+.

SUPPLEMENTAL FIGURE 5. Characterization of Boost and Boost+ grafts in unilateral 6-OHDA lesioned rat striatum

(A) Immunohistochemistry (IHC) for TH shows graft-derived dopaminergic fibers innervating the host striatum in Boost and Boost+ cell grafts. Scale bar=500 µm (left), 200 µm (right). Nuclei were counterstained with hematoxylin. **(B-C)** Representative IHC images showing TH^{+} cells co-expressing GIRK2 with CALBINDIN **(B)** or ALDH1A1 **(C)** in Boost and Boost+ grafts. Left arrowheads show TH+GIRK2-CALBIDIN- cells, while right arrows show TH+GIRK2+**(B)**. In Boost grafts, TH+CALBIDIN+GIRK2+ is shown as split channels **(B)**. Arrowheads point to TH+ GIRK2+ALDH1A1- cells, while short arrows show GIRK2+TH+ALDH1A1+ **(C)**. Long arrow indicates ALDH1A1+TH+GIRK2- **(C)**. **(D)** Representative images of IHC for human-specific Ki67 and human-specific marker hKu80 in the Boost and Boost+

grafts. Scale bar=50 µm. **(E)** Representative IHC images for human-specific COL1A1 (hCOL1A1) and hKu80 in Boost and Boost+ grafts. Scale bar=100 µm (left), 50 µm(right). **(F-G)** Representative images for hCOL1A1 and PDGFRα in Boost+ grafts. Scale bar=10 µm. **(G)** Representative images for transthyretin (Prealbumin) and hKu80. Scale bar=50 µm. **(H-O)** Whole-cell recordings from striatal spiny projection neurons (SPN) proximal to the mDA neurons graft. Examples of SPN response to current injections **(H)**, voltage-current curve **(I),** and dependence of action potential frequency on the amplitude of injected current **(J).** ****- p<0.0001 from other groups by 2-way ANOVA. Membrane properties of SPNs, including resting membrane potential (**K**, n=10-16 cells), input resistance **(L)**, membrane capacitance **(M),** and rheobase **(N)**. **(O)** Representative EPSC traces evoked by electrical stimulation of the corpus callosum in the presence of GABAA antagonist picrotoxin (**left**) and dependence of EPSC amplitude on stimulation current intensity (**right**). **** and *- different from all other groups by 2-way ANOVA with p<0.0001 and 0.05, correspondingly.

SUPPLEMENTAL FIGURE 6. KEGG-GO analysis of NURR1+ or TH+ mDA neurons comparing in vivo grafts (snRNA-seq) and age-compatible in vitro mDA neurons (scRNAseq, Day 40) in Boost and Boost+ conditions.
